# Donor embryonic stem cells displace host cells of 8-cell-stage chimeras to the extra-embryonic lineages by spatial crowding and FGF4 signalling

**DOI:** 10.1242/dev.204518

**Published:** 2025-06-25

**Authors:** Stanley E. Strawbridge, Anna Katharina Schrattel, Peter Humphreys, Kenneth A. Jones, Jérôme Artus, Anna-Katerina Hadjantonakis, Alexander G. Fletcher, Jennifer Nichols

**Affiliations:** ^1^ Cambridge Stem Cell Institute, University of Cambridge, Jeffrey Cheah Biomedical Centre, Puddicombe Way, Cambridge CB2 0AW, UK; ^2^ Department of Physiology, Development and Neuroscience, University of Cambridge, Downing Street, Cambridge CB2 3DY, UK; ^3^ Developmental Biology Program, Sloan Kettering Institute, Memorial Sloan Kettering Cancer Center, New York, NY 10065, USA; ^4^ School of Mathematical and Physical Sciences, University of Sheffield, Hicks Building, Hounsfield Road, Sheffield S3 7RH, UK; ^5^ Insigneo Institute, University of Sheffield, Sheffield S10 2TA, UK

**Keywords:** Mouse embryo, Epiblast, Trophectoderm, Primitive endoderm, Mathematical modelling, Bayesian inference

## Abstract

Following mouse embryo compaction, outer cells become trophectoderm, while inner cells form the inner cell mass (ICM), later differentiating into primitive endoderm and epiblast during blastocyst formation. Trophectoderm specification is driven by position-governed polarisation, while primitive endoderm specification is positively regulated by FGF4 signalling from the unspecified ICM and epiblast. When injected into an 8-cell-stage morula, embryonic stem cells (ESCs; derived from pre-implantation epiblast cells *in vitro*) can exclude host cells from the epiblast, leading to mice derived entirely from these cells. While evidence suggests roles for ESC-produced FGF4 and physical crowding in host cell displacement from the ICM, the interplay between these possible mechanisms has yet to be dissected, in part due to the lack of studies using *Fgf4^−/−^* ESCs. Here, we combine chimera titration assays with mathematical modelling to study these mechanisms of host cell displacement. Both *Fgf4^+/+^* and *Fgf4^−/−^* ESCs displaced host cells from the epiblast, while only *Fgf4^−/−^* ESC-injected embryos reduced primitive endoderm and increased trophectoderm, indicating sequential exclusion by displacement crowding followed by FGF4 signalling.

## Teaser

**Highlighted Article:** In mouse blastocyst chimeras, donor cells displace host cells from the epiblast by a combination of crowding and FGF4 signalling.

## INTRODUCTION

**Fig. 1.**
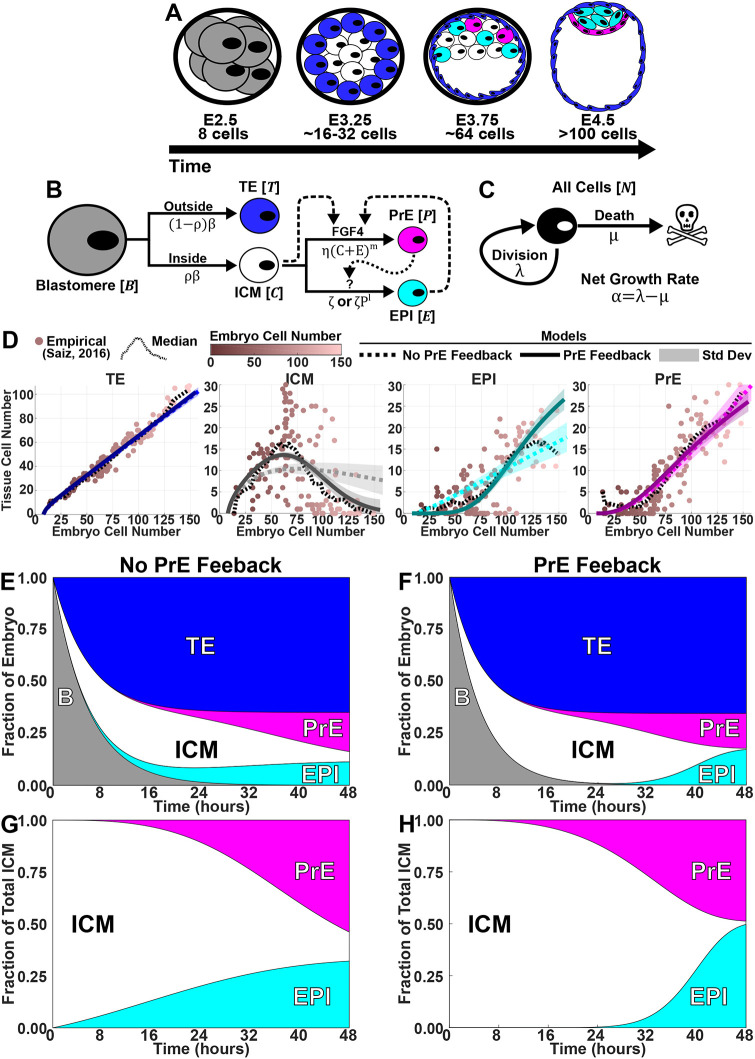
**Quantitative modelling implicates PrE feedback in ICM specification.** (A) Pre-implantation mouse development from E2.5 (8-cell morula) to E4.5 (late blastocyst). (B) Model of cell-state transitions during the first two fate decisions in mouse development. Solid lines indicate transitions; dashed lines indicate FGF4 feedback; dotted line indicates proposed PrE feedback. (C) All cells grow at net rate α. (D) Numerical solutions using median posterior parameters from models without (dotted) and with (solid) PrE feedback, overlaid on data (dots) from Saiz et al. (2016) and binned median. Shaded areas represent standard deviation. (E-H) Reconstructed time course of relative tissue sizes in whole embryo (E,F) and ICM (G,H) for models without (E,G) and with (F,H) PrE feedback.

The first fate decision in the mouse embryo is driven by positional cues, whereby outer blastomeres polarise to become the trophectoderm (TE), the founding tissue of the placenta (Fig. 1A,B). The TE forms an epithelium surrounding the bipotent inner cell mass (ICM) (Hillman et al., 1972; Tarkowski and Wróblewska, 1967), which then segregates into the primitive endoderm (PrE), the source of the yolk sac, and epiblast (EPI), which will form the future fetus and is the source of embryonic stem cells (ESCs) (Gardner and Rossant, 1979; Martin, 1981; Evans and Kaufman, 1981; Boroviak and Nichols, 2014). Specification of the ICM occurs asynchronously (Plusa et al., 2008; Saiz et al., 2016), largely due to fibroblast growth factor 4 (FGF4) secretion from unspecified ICM cells and EPI (Feldman et al., 1995; Frankenberg et al., 2011; Kang et al., 2013; Nichols et al., 2009; Yamanaka et al., 2010). Basement membrane components, including laminin 511, increase the efficiency of ESC capture (Boroviak et al., 2014), and PrE is known to express basement membrane-associated genes such as collagen 4a1 and laminin 1 (Kang et al., 2017), thus implicating PrE-produced basement membrane in EPI establishment and/or maintenance.

The dynamic allocation of the three lineages can be perturbed in myriad ways, such as re-incorporating ESCs into pre-implantation embryos by injection or aggregation to form chimeras (Poueymirou et al., 2007; Grabarek et al., 2012; Humięcka et al., 2016). These donor cells can re-enter normal development from the pre-implantation EPI stage and can contribute to all adult germ layers and the germ line (Bradley et al., 1984). Injecting donor ESCs into 8-cell embryos at embryonic day (E) 2.5 can increase TE cell numbers by displacing host blastomeres outward (Fig. 1B) (Humięcka et al., 2016). Donor cells can also increase host-derived PrE cell numbers. With sufficient donor cells, the resulting mouse may be entirely donor derived; in contrast, blastocyst-stage injection yields chimeras that are only partially derived from ESCs (Poueymirou et al., 2007). This modulation in the second fate decision likely stems from FGF4 production by donor ESCs. Indeed, high exogenous FGF4 levels can drive the entire specifying ICM to the PrE fate (Yamanaka et al., 2010). While FGF4 is a key driver of ICM specification, no feedback role has been proposed for PrE. However, laser ablation studies show that the specifying ICM will compensate for EPI or PrE loss (Saiz et al., 2020), suggesting population-level feedback between these three cell types.

Here, we unite these observations into a theoretical framework. We first generated a compartment model of cell population dynamics in the E2.5-E4.5 mouse embryo, calibrated against previous observations (Saiz et al., 2016), indicating a role for PrE feedback on ICM specification. Using this model, we conducted donor cell injections into host embryos using wild-type (WT; *Fgf4^+/+^*) and *Fgf4^−/−^* ESCs. Both donor types impeded host EPI contribution, with a smaller PrE and larger TE observed in the *Fgf4^−/−^* case. Finally, we combined our base model with chimera assays to model chimera formation, indicating that donor cells perturb host cell allocation by spatial crowding and subsequent FGF4 induction.

## RESULTS AND DISCUSSION

### A compartment model of blastocyst generation suggests a role for feedback from the PrE on ICM specification

Previous models of blastocyst generation have focused on position for the first fate decision and a bistable gene regulatory network for ICM specification (Bessonnard et al., 2014; Nissen et al., 2017; Saiz et al., 2020). Here, we aimed to create a minimal model of blastocyst formation, extendable to generate *in silico* chimeras by adding donor ESCs. This model provides outputs for transition rates and numbers and proportions of cells in each lineage. We designed a compartment model of mouse embryogenesis spanning the E2.5 8-cell stage to the E4.5 late blastocyst stage (Fig. 1B). During this period, two binary cell-fate decisions occur. First, blastomeres, *B*, specify, at a rate β, into unspecified ICM cells, *C*, with a bias of ρ, or into TE, *T*, with a bias of 1−***ρ***. Second, unspecified ICM cells become either PrE, *P*, or EPI, *E*. PrE specification is driven by FGF4 secreted from the unspecified ICM cells and EPI. This is reflected in the PrE specification rate as *η* (*C*+*E*)^*m*^, where *η* is a constant and *m* is a feedback parameter to allow for potential non-linearity. For EPI specification, we compare two models. The first is a constant rate of specification, *ζ*. This emulates one school of thought that the unspecified ICM will take on the EPI identity by default in the absence of FGF4. Our alternative model is undetermined feedback from the PrE, possibly involving extracellular matrix components, such as laminin. In this model, the specification rate is *ζ P*^*l*^, where *l* is a feedback parameter to allow for potential non-linearity. Finally, for simplicity we assumed that all cells proliferate with the same net growth rate α, which captures both cell division and death (Fig. 1C). Partial evidence in support of this simplifying assumption is provided by a good linear fit between TE and non-TE cell numbers in our data (Fig. S1A).

We inferred parameters for both models using an approximate Bayesian computation with Markov chain Monte Carlo (ABC-MCMC), with data from Saiz et al. (2016) (Fig. 1D, Fig. S1B,C). Both models yielded net growth rates around 0.06 h^−1^, corresponding to a doubling time of ∼11 h. This rate allows an 8-cell morula to undergo four doublings, producing ∼128 cells. Both models also capture the TE (65%) to unspecified ICM (35%) ratio, with an unspecified ICM bias ***ρ***=0.35. To assess the sufficiency of no, linear or nonlinear feedback, parameters *l* and *m* were varied among 0, 1 and 2. In all cases, the inference algorithm selected a nonlinear feedback (value of 2). Both models reproduced TE and PrE proportions well (Fig. 1E-H), but the model without PrE feedback failed to capture the dynamics of unspecified ICM and EPI. Since the ICM both generates and responds to FGF4, we hypothesise that PrE specifies earlier than EPI. Supporting this, Saiz et al. (2016) show more early PrE than EPI cells. However, Plusa et al. (2008) and Grabarek et al. (2012) report that EPI plasticity is lost before PrE. A Bayes factor of ∼17 (based on 500 acceptances from 6289 and 105,027 parameter sets, for the PrE feedback model and non-feedback model, respectively, with ε=17,300) provides positive evidence in favour of the PrE feedback model over the non-feedback model (Toni et al., 2009). This is especially pronounced when considering unspecified ICM, which should be fully resolved into EPI or PrE by E4.5 (Fig. 1D,G,H). To capture intrinsic stochasticity in small cell populations, we ran Gillespie simulations using the median posterior parameter values from the PrE feedback model (Fig. S1D). These reproduced observed cell type distributions, validating parameter estimates and confirming that the deterministic model approximates cell population dynamics well.

This suggests that PrE feedback is sufficient to drive timely ICM specification. While we favour the hypothesis that induction of ICM to EPI is mediated by basement membrane components from the PrE, this remains unproven. Further experimental and theoretical work is needed to clarify the mechanism. Next, we expanded on our base model to examine how donor cells perturb host cell allocation, generating two datasets to explore host–donor interactions and tissue modulation in response to donor cells.

### Both *Fgf4^+/+^* and *Fgf4^−/−^* donor ESCs can impede host cells from contributing to the EPI

**Fig. 2.**
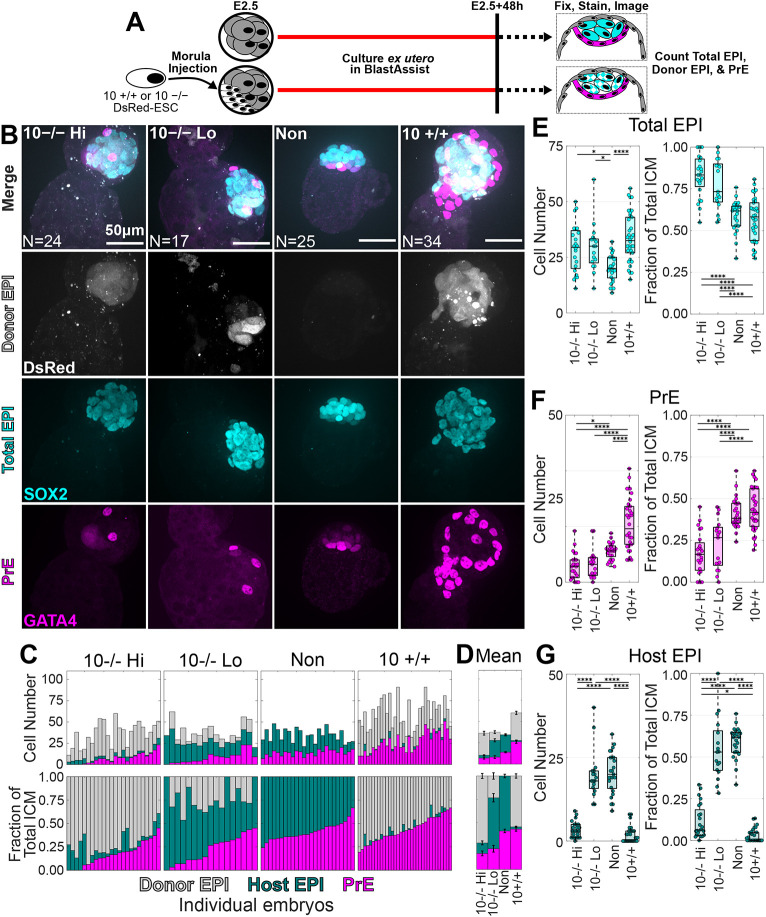
**Both *Fgf4^+/+^* and *Fgf4^−/−^* donor ESCs can impede host contribution to the EPI.** (A) Schematic of ICM imaging after injection of ten donor cells. (B) Representative maximum intensity projections of confocal *z*-stacks for chimeras injected with ten *Fgf4^−/−^* ESCs showing high (Hi; *n*=24) or low (Lo; *n*=17) donor contribution, non-injected embryos (*n*=25) and ten *Fgf4^+/+^* ESC-injected chimeras (*n*=34). (C,D) Stacked bar plots showing individual (C) and mean±s.e.m. (D) ICM composition by cell number (top) and fraction (bottom). Magenta, GATA4^+^ PrE; dark green, SOX2^+^ host EPI; grey: DsRed^+^/SOX2^+^ donor cell-derived EPI. Bars sorted by PrE fraction. (E-G) Box/swarm plots showing cell number (left) and ICM fraction (right) for total EPI (E), PrE (F) and host-derived EPI (G). Box plots show the minimum, first quartile (Q1), median (Q2), third quartile (Q3) and maximum values, with the box representing the interquartile range (Q1−Q3), the line inside the box indicating the median, and whiskers extending to the most extreme values. The dots represent individual data points. *0.05≥*P*>0.01; ****0.0001≥*P* (pairwise comparisons by N-way ANOVA). See Table S1 for full summary statistics and exact *P*-values.

Previous work has shown that donor ESCs have the ability to displace host cells from EPI through FGF4 signalling (Poueymirou et al., 2007; Humięcka et al., 2016). To disentangle FGF4 signalling from any other mechanism, we investigated host EPI displacement by injecting either ten *Fgf4^+/+^* or ten *Fgf4^−/−^* ESCs into WT 8-cell stage morulae, via perforation of intact zona pellucida (Fig. 2A). Chimeric embryos were cultured *ex utero*, alongside WT non-injected embryos, for 48 h to the late blastocyst stage. Embryos were then fixed, immunostained and imaged for the EPI marker SOX2, the PrE marker GATA4 and the donor cell marker DsRed (Fig. 2B). ICM cell numbers and types were determined manually with the ImageJ plugin ‘Cell Counter’ (Fig. 2C,D). Two groups with varying levels of chimerism were observed in the ten *Fgf4^−/−^* ESC-injected (10^−/−^) embryos. This classification was supported by k-means clustering, which yielded an optimal silhouette score of 0.6252, indicating moderate structure consistent with two biologically distinct subpopulations (Fig. S2A-C) (Kaufman and Rousseeuw, 1990). The two resulting groups were chimeras with high (Hi) and low (Lo) levels of donor cell contribution to the total EPI.

All three groups of chimeras had EPIs with greater cell numbers than non-injected embryos (Fig. 2E). However, when examining the EPI fraction of the ICM, only 10^−/−^ Hi and Lo chimeras had larger EPI compartments. The reason for this became apparent upon inspecting the number of PrE cells within the different chimera conditions (Fig. 2F). Both the 10^−/−^ Hi and 10^−/−^ Lo chimeras showed a reduced PrE cell number and fraction of the ICM, while *Fgf4^+/+^* ESC-injected (10^+/+^) chimeras showed a reciprocal increase in both PrE cell number and fraction. Finally, 10^+/+^ and 10^−/−^ Hi chimeras had fewer host cells in their EPI compared to the 10^−/−^ Lo chimeras and non-injected embryos (Fig. 2G).

This suggests alternative mechanisms of host EPI exclusion by *Fgf4^+/+^* and *Fgf4^−/−^* donor cells. In 10^+/+^ chimeras, we propose that FGF4^+/+^ donor ESCs drive host unspecified ICM cells into the PrE via sustained FGF4 signalling, effectively excluding host cells from the EPI. In contrast, *Fgf4^−/−^* donor cells lack FGF4 production, implying a different exclusion mechanism. Are potential host EPI cells lost through reduced proliferation or apoptosis, or are they, as in 10^+/+^ chimeras, redirected to another lineage such as TE? To investigate, we next quantified cell number and lineage identity in late blastocysts.

### Embryos injected with *Fgf4^−/−^* donor ESCs have fewer PrE and more TE cells

**Fig. 3.**
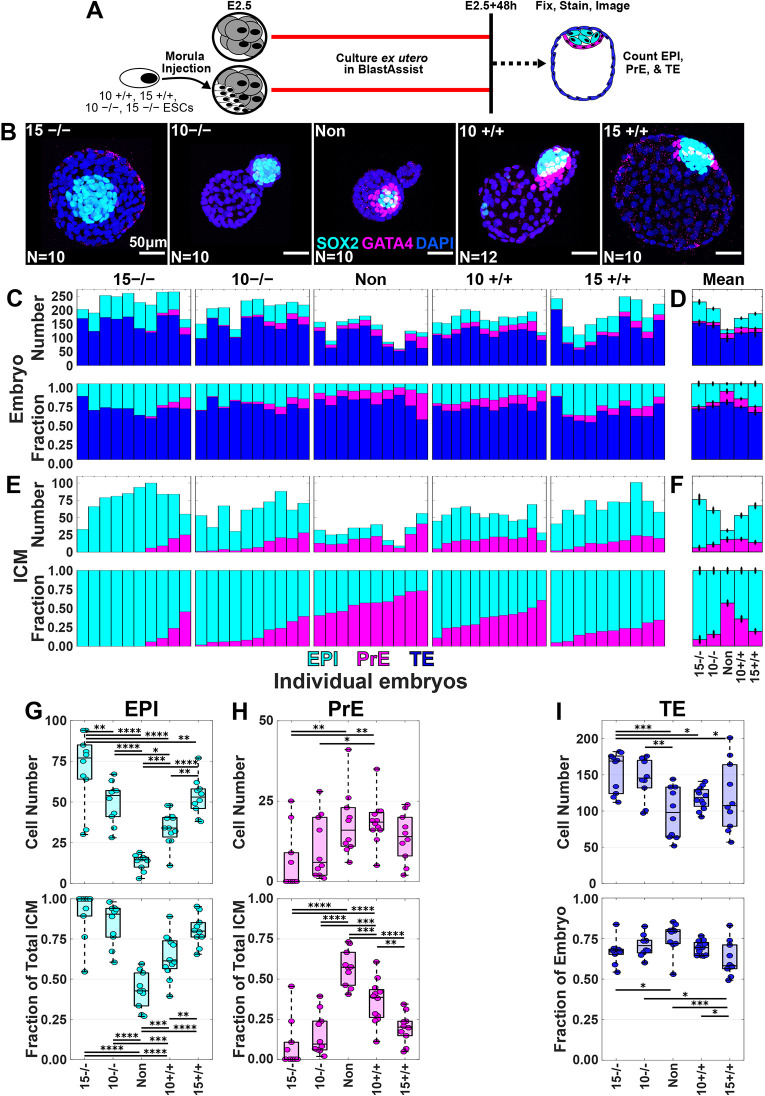
**Embryos injected with *Fgf4^−/−^* donor ESCs have fewer PrE cells and more TE cells.** (A) Schematic of whole-embryo imaging after injection of ten or 15 donor cells. (B) Representative maximum intensity projections of confocal *z*-stacks for non-injected embryos (*n*=10), ten and 15 *Fgf4^−/−^* ESC-injected chimeras (*n*=10,10), and ten and 15 *Fgf4^+/+^* ESC-injected chimeras (*n*=12,10). (C,D) Stacked bar plots of individual (C) and mean±s.e.m. (D) data. ICM composition for chimeras and embryos showing cell number (top) and whole-embryo fraction (bottom). Magenta, GATA4^+^ PrE; cyan, SOX2^+^ EPI; blue, double-negative TE. Bars sorted by PrE fraction. (E,F) Stacked bar plots for individual (E) and mean±s.e.m. (F) data. ICM composition for chimeras and embryos showing cell number (top) and fraction of the ICM (bottom). (G,H) Box/swarm plots showing cell number (top) and ICM fraction (bottom) for EPI (G) and PrE (H). (I) Box/swarm plots showing cell number (top) and whole-embryo fraction (bottom) for TE. Box plots show the minimum, first quartile (Q1), median (Q2), third quartile (Q3) and maximum values, with the box representing the interquartile range (Q1−Q3), the line inside the box indicating the median, and whiskers extending to the most extreme values. The dots represent individual data points. *0.05≥*P*>0.01; **0.01≥*P*>0.001; ***0.001≥*P*>0.0001; ****0.0001≥*P* (pairwise comparisons by N-way ANOVA). See Table S1 for full summary statistics and exact *P*-values.

We investigated how *Fgf4^−/−^* donor ESCs perturb host cell allocation by quantifying cell numbers of all three lineages at the late blastocyst stage (Fig. 3A). We injected ten or 15 *Fgf4^+/+^* (15^+/+^) or *Fgf4^−/−^* (15^−/−^) ESCs into WT 8-cell stage morulae. Chimeric and non-injected WT embryos were cultured for 48 h to the late blastocyst stage, then fixed, stained for DAPI (nuclei), SOX2 (EPI) and GATA4 (PrE), and imaged (Fig. 3B). Cells were segmented and cell types determined as described in Materials and Methods (Fig. 3C-F).

Consistent with the previous experiment, all chimera conditions showed increased EPI cell number and ICM fraction compared to non-injected embryos (Fig. 3G), with EPI scaling proportionally to the number of donor cells, regardless of type. Conversely, PrE cell number and ICM fraction were reduced in 10^−/−^ and 15^−/−^ chimeras (Fig. 3H), consistent with prior findings that *Fgf4^−/−^* donor ESCs impede PrE specification. Compared to earlier 10^+/+^ chimeras, both 10^+/+^ and 15^+/+^ chimeras showed reduced PrE fractions, with 15^+/+^ exhibiting a greater decrease. Overall, higher numbers of donor cells, regardless of genotype, consistently lowered the PrE fraction within the ICM.

In *Fgf4^−/−^* ESC-injected embryos, TE cell numbers increased reciprocally with a decrease in PrE cells (Fig. 3I). The proportions of 15^+/+^ and 15^−/−^ chimeras composed of TE cells were significantly smaller than in non-injected embryos, likely due to increased EPI cell numbers. These findings suggest that donor cells, regardless of genotype, can drive host cells into the TE compartment during the first cell-fate decision. In chimeras with *Fgf4^−/−^* donor cells, remaining host ICM cells can specify into either EPI or PrE. In contrast, in chimeras with *Fgf4^+/+^* donor cells, these unspecified host ICM cells are biased toward PrE, likely via FGF4 signalling. We next formalise these concepts in a mathematical model of chimera formation.

### Quantitative modelling suggests a role for displacement crowding and FGF4 signalling in host EPI exclusion

**Fig. 4.**
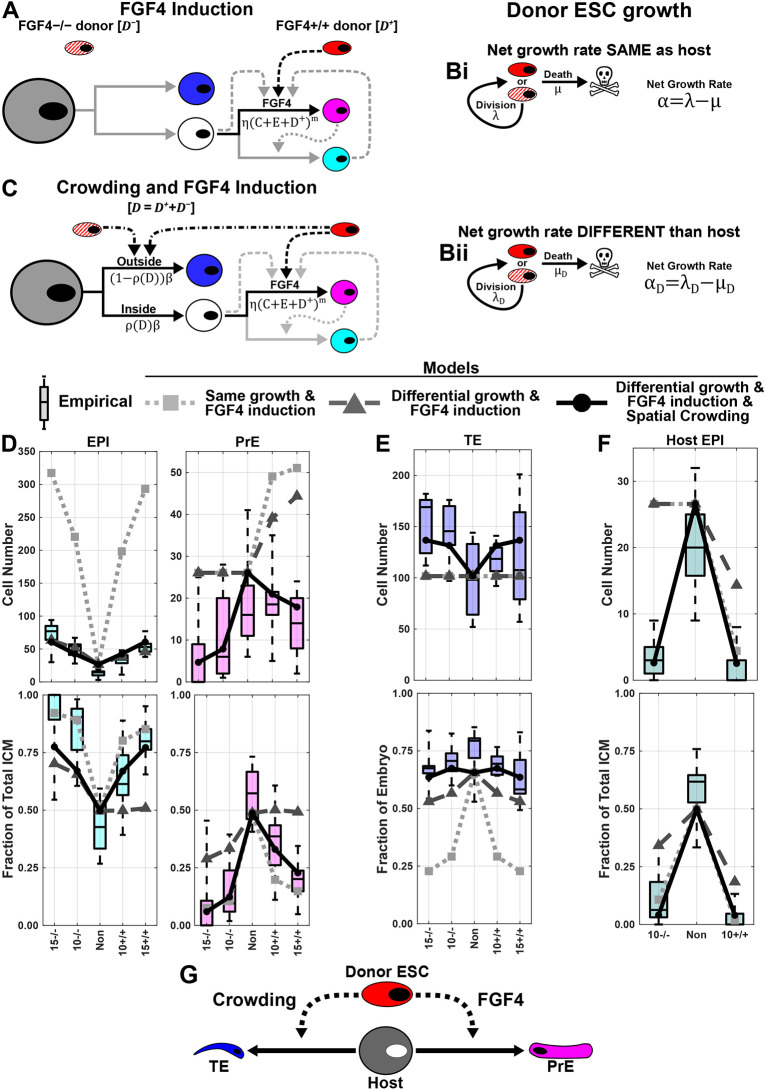
**Quantitative modelling suggests spatial crowding plays a role in host EPI exclusion during chimera formation.** (A,C) FGF4 induction model (A) and FGF4 induction and spatial crowding model (C) for chimera formation. Grey, blastomere; blue, TE; white, unspecified ICM; cyan, EPI; magenta, PrE; red, *Fgf4^+/+^* donor ESC; red/white, *Fgf4^−/−^* donor ESC. Solid lines indicate cell-state transitions, dashed lines FGF4 feedback, dotted line posited PrE feedback, and dash-dotted line spatial crowding feedback. (B) Donor ESC net growth rate: (i) fixed at host cell rate; (ii) inferred. (D,E) Simulations (lines and markers, see key) superimposed on data from Fig. 3G-I used to infer additional parameters. (F) Simulations superimposed on host EPI data from Fig. 2G. (G) Proposed host EPI exclusion model.

We integrated donor cells into our base model of embryogenesis to generate *in silico* chimeras and compared three models of chimera formation. The first model includes FGF4 induction from *Fgf4^+/+^* donor cells, *D*^+^ (Fig. 4A), and both *Fgf4^+/+^* and *Fgf4^−/−^* donor cells, *D*^−^, have the same net growth rate as host cells (F) (Fig. 4Bi). The second model includes FGF4 induction and a different net growth rate, differential growth (GF) (Fig. 4Bii), and the third model includes FGF4 induction, differential growth, and spatial crowding, which drives host cells towards the TE, from both types of donor cells (GFC) (Fig. 4C). These models were simulated using the posterior median values for parameters from the base model of embryogenesis, including the host cell net growth rate (Fig. 1B,C). Additional parameters for the GF and GFC models were estimated by ABC-MCMC using data from Fig. 3. Donor cell net growth rate, α_D_, was determined for the GF model and donor cell net growth rate, crowding factor, *a*, and feedback parameter, *n*, were estimated for the GFC model. Models were simulated using the median of the posterior parameter distribution (Fig. 4D-F, Fig. S3A,B). The inferred donor growth rate (*α*_*D*_) was approximately 0.02 h^−1^, consistent with empirical observations of ∼0.018 h^−1^ based on live-cell tracking of cells derived from donor ESCs in chimeric embryos (Alexandrova et al., 2016).

Upon examining model performance, we saw the F model had EPI cell numbers more than five times larger than observed (Fig. 4D), while both GF and GFC models better matched EPI cell numbers. However, the GF model performed poorly against 10^+/+^ and 15^+/+^ chimeras with low EPI fractions of the ICM. Neither F nor GF models performed well against the PrE cell numbers, overestimating the values in all observed chimeras. The GFC model, by contrast, followed the PrE trends in both cell number and fraction of the ICM. When considering the TE, the F and GF models showed no modulation in TE cell number whereas the GFC model does (Fig. 4E). Finally, we tested the predictive power for these models against data they have not been exposed to (Fig. 4F). We found that only the GFC model predicts both host EPI cell numbers and fraction of the ICM. Neither the F nor the GF model is able to predict host EPI exclusion in terms of cell number for the 10^−/−^ chimeras. The Bayes factor between the GFC model and FC model was around 45 (based on 500 acceptances from 585 and 26,575 parameter sets, respectively, using a rejection ABC threshold of ε=117,000), providing strong evidence in favour of the GFC model over the FC model (Toni et al., 2009). To assess further how well the GFC model reflects biological variability in small cell populations, we performed stochastic simulations using the median posterior parameter values (Fig. S3C). These simulations reproduced both the range and variability of lineage contributions seen in the experimental data, suggesting that the model captures key sources of noise and heterogeneity in chimera development. This suggests that the donor cells perturb host tissues through spatial crowding in the first cell-fate decision and FGF4 induction in the second cell-fate decision (Fig. 4G).

It has been shown that the ESCs sort to the interior of the E2.5 8-cell stage embryo, while blastomeres remain on the exterior (Humięcka et al., 2016). The mechanisms underlying this process have not yet been established, but could include differential cell contractility (Maître et al., 2016) or membrane fluctuations (Yanagida et al., 2022). As a result, some host cells that would otherwise be located to the interior may be forced to the exterior and specified as TE. For *Fgf4^−/−^* donor ESCs, the remaining host ICM cells specify normally as either EPI or PrE, whereas for *Fgf4^+/+^* donor ESCs, host cells show a bias toward PrE. Taken together, these findings advance our understanding of how donor ESCs influence lineage allocation of host cells as ESCs reincorporate into normal development, shedding light on mechanisms of cell-fate plasticity in the early mouse embryo.

## MATERIALS AND METHODS

### ESC culture

*Fgf4^+/+^* and *Fgf4^−/−^* ESCs from the CD1 background (Wilder, 1997) were cultured in 2i/Lif (Ying et al., 2008) in accordance with established protocols (Mulas et al., 2019). Donor cells used in chimera experiments were labelled with dsRES via 1 μg pPB-CAG-dsRED-pgk-Hyg (Guo et al., 2009), co-transfected with 2 µg of transposase (pPBase; Wang et al., 2008), using Lipofectamine 2000. The cells were plated onto hygromycin-resistant feeders and selected with 200 µg/ml hygromycin after 48 h. Individual colonies were picked after 14 days based upon dsRed expression levels.

### Embryo culture and chimera generation

Embryos were obtained from natural mating (C57BL/6xCBA). Detection of a copulation plug in the morning was used as confirmation of successful mating and designated E0.5. Embryos were flushed from oviducts at E2.5 (8-cell stage) using M2 (Sigma-Aldrich, M7167). Optimally, for chimera formation, embryos would be at the uncompacted 8-cell stage, enabling donor cells injected through the zona pellucida to become incorporated within the morula as it compacts. Embryo stages can vary within and between litters. Those recovered at the 4-cell stage were cultured to the 8-cell stage. Embryos that had already compacted were decompacted by brief culture in calcium-free medium for a few minutes before injection. Occasional abnormal embryos were discarded, but otherwise all embryos were used. For the injection procedure, embryos and donor cells were placed in drops of M2 under oil on the microscope stage of the injection rig. Embryos were immobilised by means of a suction-mediated holding pipette. The desired number of separated ESCs were aspirated into the injection pipette. A small hole, just big enough to insert the injection pipette, was made in the zona pellucida opposite the holding pipette in a region of maximal space between blastomeres using a XYClone laser (Hamilton Thorne Biosciences). The injection pipette was gently pushed through the hole in the zona and the donor cells deposited away from the hole, between zona and host cells. Embryos were subsequently cultured for 48 h in BlastAssist (Origio) as either a control or following ESC injection (Poueymirou et al., 2007). The experiments in Figs 2 and 3 were conducted independently, on different days, and by different operators. Specifically, the injections in Fig. 2 were performed by a more experienced operator, while those in Fig. 3 were performed by a trainee. Reduced cell viability in Fig. 3 may have contributed to the lower efficiency of chimera formation, likely due to the longer injection times associated with training. This could explain both the subtle shifts in lineage proportions and the observed reduction in PrE contribution in the 10^+/+^ group in Fig. 3 that is not seen in Fig. 2.

This research has been regulated under the Animals (Scientific Procedures) Act 1986 Amendment Regulations 2012 following ethical review by the University of Cambridge Animal Welfare and Ethical Review Body. Use of animals in this project was approved by the ethical review committee for the University of Cambridge, and relevant Home Office licences (Project licence number 80/2597 and number P76777883) were in place.

### Immunohistochemistry

Embryos and chimeras were cultured to E2.5+48 h post-harvest and fixed in 4% paraformaldehyde in PBS for 15 min. They were rinsed in PBS with 3 mg/ml polyvinylpyrrolidone and blocked in 2% donkey serum, 0.01% bovine serum albumin, 0.01% Tween 20 in PBS for ∼15 min. Primary antibodies were rat monoclonal anti-SOX2 (eBioscience, 14-9811-80) at 1:500, goat polyclonal anti-GATA4 (Santa Cruz Biotechnology, SC-1237) at 1:400, and DAPI at 1:10,000 in blocking buffer. Embryos were incubated in primary antibodies at 4°C overnight, then rinsed three times in blocking buffer for at least 15 min each. Secondary antibodies conjugated with Alexa Fluor dyes of appropriate fluorophores and raised against the required hosts [donkey anti-rat Alexa Fluor 488 (Thermo Fisher Scientific, A-21208) or donkey anti-goat Alexa Fluor 647 (Thermo Fisher Scientific, A-21447)] were diluted 1:500 in blocking buffer and embryos incubated for 1-2 h at room temperature in the dark. They were subsequently rinsed as previously, equilibrated through increasing concentrations of mounting buffer (Vectashield; Vector Laboratories, H-1200), transferred to drops of concentrated mounting buffer on slides under coverslips subsequently sealed with nail varnish. Confocal images were acquired using an Andor Revolution XD spinning disc confocal microscope and Leica SP5 laser scanning microscope.

### Quantitative image analysis

Manual cell counting of ICMs from embryos and chimeras collected at E2.5+48 h was performed on confocal *z*-stacks using Fiji (ImageJ) with the ‘Cell Counter’ plugin. Individual nuclei were scored and assigned to lineages by eye, based on fluorescence marker expression. A nucleus was considered marker-positive if its signal was clearly above local background and showed nuclear enrichment for SOX2 and GATA4, or both nuclear and cytosolic enrichment for DsRed. SOX2-positive cells were classified as EPI, GATA4-positive cells as PrE, and cells double-positive for DsRed and SOX2 as donor-derived EPI. Semiautomated cell counting of whole embryos and chimeras collected at E2.5+48 h was performed with the MATLAB-based algorithm MINS (Lou et al., 2014) to perform 3D nuclear segmentation of the DAPI channel. Post analysis corrections for over- and under-segmentation was performed by manual segmentation in Fiji (Schindelin et al., 2012) using ROImanager to quantify (*x*,*y*,*z*) coordinates and fluorescence intensity for DAPI, SOX2 and GATA4. Each segment was sorted into SOX2 single-positive, GATA4 single-positive, or SOX2/GATA4 double-negative populations by agglomerative hierarchical clustering using MATLAB (2016B) LINKAGE with WARD distances and the CLUSTER function. Individual segments were then clustered into 3D nuclei. Optimisation of the clustering and number of nuclei was performed by Scree analysis and the elbow method, respectively. Nuclei that were segmented correctly by MINS were clustered into TE, PrE and EPI in the same manner as the initial segment sorting (Strawbridge et al., 2023). Clustering was performed on a per-embryo basis.

### Mathematical modelling

We modelled the mouse embryo cell population dynamics from the 8-cell stage (E2.5) to the late blastocyst stage (E4.5). Our mean-field model comprises a set of coupled ordinary differential equations (ODEs), which reflect our assumptions regarding cell-state transitions, proliferation and death (Fig. 3A,B). These equations govern the evolution in time (*t*) of the numbers of blastomeres (*B*), TE cells (*T*), unspecified ICM cells (*C*), PrE cells (*P*), EPI cells (*E*), and – for chimeric embryos (Fig. 4A-C) – the numbers of donor ESCs that are *Fgf4^+/+^* (*D*^+^) or *Fgf4^−/−^* (*D*^−^).

We assumed that all host cells proliferate with constant net per-capita rate *α*, which captures the net effect of division and death on cell number. This simplifying assumption is supported by a strong linear relationship between TE and ICM-derived cell numbers (unspecified ICM, PrE and EPI), with a standardised effect size of *β*=1.1143, suggesting that these lineages expand at comparable rates during this developmental window (Fig. S1A). We modelled the sorting of blastomeres to the interior of the embryo, where they form the unspecified ICM, and exterior, where they epithelialise and become TE, as irreversible cell-state transitions with constant per-capita rates *ρβ* and (1−*ρ*)*β*, respectively. Here *β* denotes a constant overall per-capita rate of blastomere differentiation and *ρ* denotes the blastomere lineage bias towards the unspecified ICM. We modelled the specification of ICM into PrE and EPI as irreversible transitions. To account for the production by PrE of extracellular matrix proteins that help drive EPI specification, we assumed that the per-capita rate of transition from unspecified ICM to EPI, *ζP*^*l*^, depends on the number of PrE cells present, with the parameter *l* allowing for non-linearity. Similarly, to account for the production by unspecified ICM and EPI of FGF4 that helps drive PrE specification, we assumed that the per-capita rate of transition from unspecified ICM to PrE, *η*(*C*+*E*)^*m*^, depends on the numbers of unspecified ICM and EPI cells present, with the parameter *m* allowing for non-linearity. Here, *ζ* captures the strength of feedback from PrE onto EPI specification, while *η* captures the strength of feedback from unspecified ICM and EPI onto PrE specification.

Accounting for the above processes leads to the following ODE system (Fig. 1A):
(1)

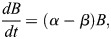

(2)

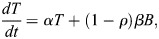

(3)

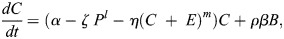

(4)

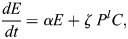

(5)

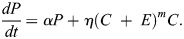
Since our model describes the cell population dynamics starting from the 8-cell stage morula, comprising only blastomeres, we impose the initial conditions *B*(0)=8,*T*(0)=*C*(0)=*E*(0)=*P*(0)=0. Our period of interest ends at *t*=48 h.

Chimera formation involves the injection of donor ESCs that are *Fgf4^+/+^* (*D*^+^) or *Fgf4^−/−^*((*D*^−^). This introduces additional contributions to the cell population dynamics. First, we assumed that donor cells proliferate at per-capita rate, *α*_*D*_, which we allow to differ from that of host cells. Second, based on previous observations (Humięcka et al., 2016), we assumed that donor cells bias the rate of transition of blastomeres to unspecified ICM by crowding displacement, leading us to replace the parameter *ρ* with the decreasing function *ρ*_0_/(1+*a*(*D*^+^+*D*^−^)). We write the sum *D*^+^+*D*^−^ for convenience here; in practice, we only considered a model in which either *Fgf4^+/+^* or *Fgf4^−/−^* ESCs are present. Third, to account for the production by *Fgf4*^+/+^ ESCs of FGF4 that helps drive PrE specification, we modified the per-capita rate of transition from unspecified ICM to PrE from *η*(*C*+*E*) to *η* (*C*+*E*+*D*^+^). Accounting for these additional processes led to the following ODE system describing cell population dynamics during chimera formation (Fig. 1C):
(6)

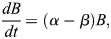

(7)

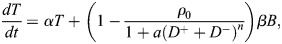

(8)

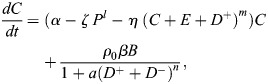

(9)

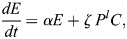

(10)

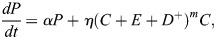

(11)

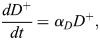

(12)

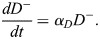
We assumed that injection of donor ESCs occurs at the 8-cell stage morula, hence imposed the initial conditions *B*(0)=8, *T*(0)=*C*(0)=*E*(0)=*P*(0)=0, and either *D*^+^ (0)=10, *D*^−^ (0)=0 (injection of ten *Fgf4*^+/+^ ESCs), *D*^+^ (0)=15, *D*^−^ (0)=0 (injection of 15 *Fgf4^+/+^* ESCs), *D*^+^ (0)=0, *D*^−^ (0)=10 (injection of ten *Fgf4^−/−^* ESCs), or *D*^+^ (0)=0, *D*^−^ (0)=15 (injection of 15 *Fgf4^−/−^* ESCs). Once again, our period of interest ends at *t*=48 h.

Eqns 1-5 and 6-11 were solved numerically in MATLAB using an explicit Runge–Kutta method; see the ‘Data and resource availability’ section for details on how to download our code.

Most of our model parameters cannot be directly measured and instead must be inferred from our data. We estimated parameters using approximate Bayesian computation (ABC) (Toni et al., 2009; Liepe et al., 2014), a likelihood-free method that iteratively compares a summary statistic from model simulations with given parameter values to the corresponding summary statistic from our data, and accepts those parameter values if these summary statistics are sufficiently close. By building up a set of accepted parameter values, ABC approximates their posterior distribution, allowing us to quantify our uncertainty in their values given our data. There are various well-established adaptations of ABC; we used a Markov chain Monte Carlo approach (Marjoram et al., 2003). We used the summary statistic

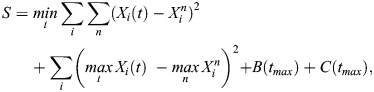
where *X*_*i*_(*t*) denotes the value of the *i*th model variable at time *t* so that *X*_1_(*t*)=*B*(*t*), *X*_2_(*t*)=*C*(*t*) and so on; 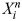 denotes the value of the *n*th observation of the *i*th variable; and *t*_*max*_ denotes the final time point in our model solution. The first term in *S* reflects our wish to have the model solution lie as close as possible to our data, accounting for the fact that time is implicit in our experimental observations (in other words, we are fitting the model in state space rather than as a time series). The second term in *S* reflects our wish to have the maximum value attained by each component of our model solution to be as close as possible to the corresponding maximum observation, as a way of helping to pin down timescales in our model. The third term in *S* reflects our wish to have as few as possible blastomeres and unspecified ICM cells present at the final time point in our model solution.

## Supplementary Material



10.1242/develop.204518_sup1 Supplementary information

Table S1. Summary of statistical analyses corresponding to Figures 2E–G and 3G–I. Summary statistics (mean, standard deviation, and N) and p-values from N-way ANOVA with Tukey's honestly significant difference (HSD) post hoc test are provided for each experimental condition. Statistical significance was assessed at α = 0.05.
