# Supplementary material for: Donor embryonic stem cells displace host cells of 8-cell-stage chimeras to the extra-embryonic lineages by spatial crowding and FGF4 signalling
Source: Development. 2025 Jun 25;152(12):dev204518. doi: 10.1242/dev.204518 (PMC12273629; doi:10.1242/dev.204518)
Supplement: Supplementary information [file develop-152-204518-s1.pdf]

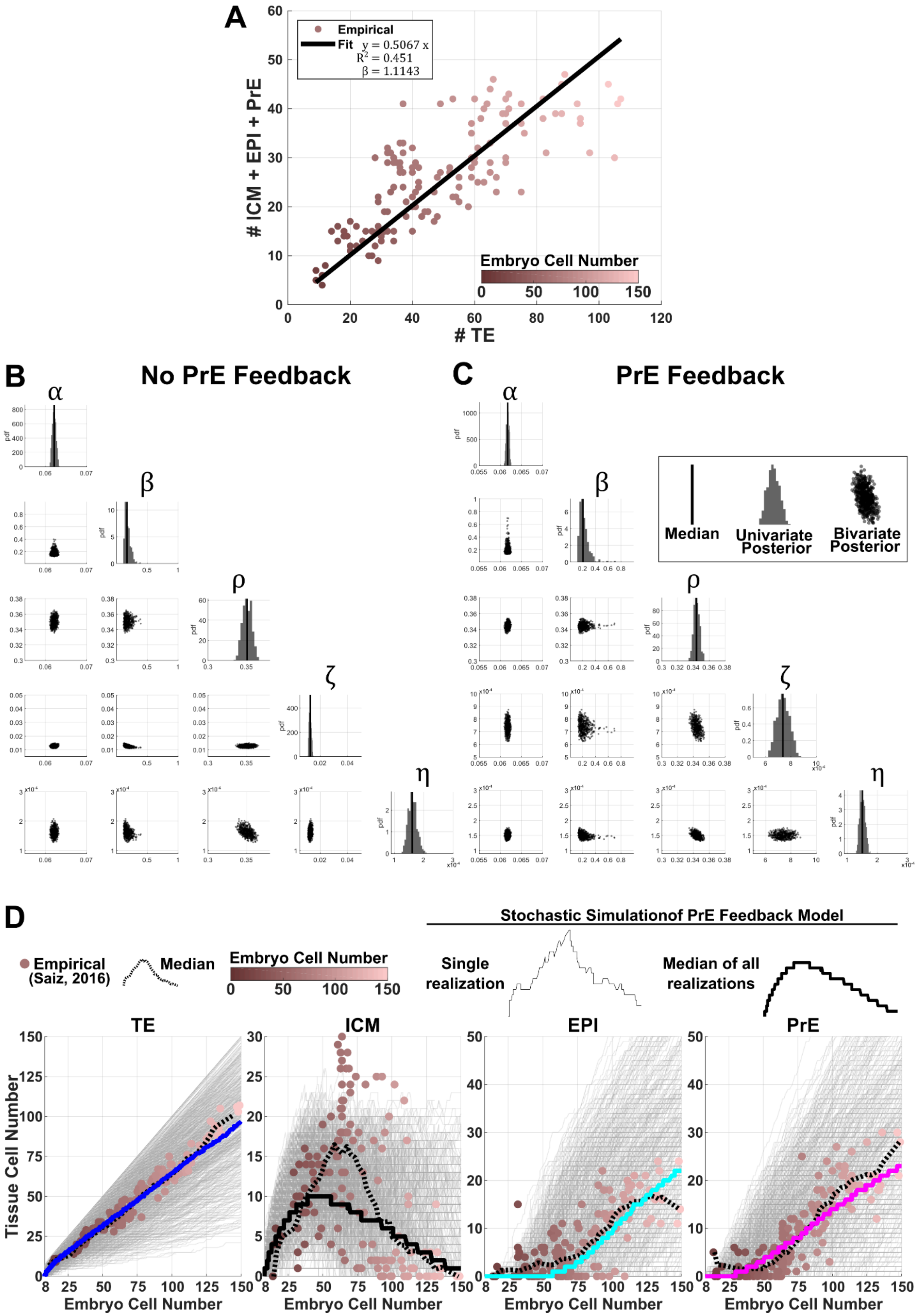

Development • Supplementary information

Fig. S1. Posterior parameter distributions and stochastic simulations for embryogenesis. A. Scatter plot showing the relationship between the number of TE and number of cells in the ICM compartment (unspecified ICM + EPI + PrE) per embryo data from (Saiz et al. 2016). A linear model was fit without intercept yielding $y = 0.5067x$, with $R^2 = 0.4510$, p-value $= 2.436 \times 10^{-80}$, and Standardized effect size $\beta = 1.1143$. Each point is colored by total embryo cell number. **B,C.** Univariate (on diagonal histograms, vertical line shows median) and bivariate (off diagonal scatter plots) posterior distributions of inferred parameters for models without (**B**) and with (**C**) PrE feedback on ICM specification. Parameters are: $\boldsymbol{\alpha}$, net growth rate; $\boldsymbol{\beta}$, blastomere specification rate; $\boldsymbol{\rho}$ blastomere bias towards the unspecified ICM fate; ζ, coefficient for ICM to EPI specification; and $\boldsymbol{\eta}$, coefficient for ICM to PrE specification. Not shown are posteriors for feedback parameters l, m, which both take on the value of 2 for all accepted parameter sets. Priors were uniform over the ranges shown in the plots. ABC thresholds (ε) were 17,300 for the model without PrE feedback and 13,500 for the model with PrE feedback. **D.** Stochastic simulations of the embryo model using the Gillespie algorithm, run with the posterior median parameters from the PrE feedback model. Shown are 1,000 independent realizations (light gray line) and median of all realizations (think colored line), overlaid on empirical data (dots) from (Saiz et al. 2016) and the median (50 bins).

Development • Supplementary information

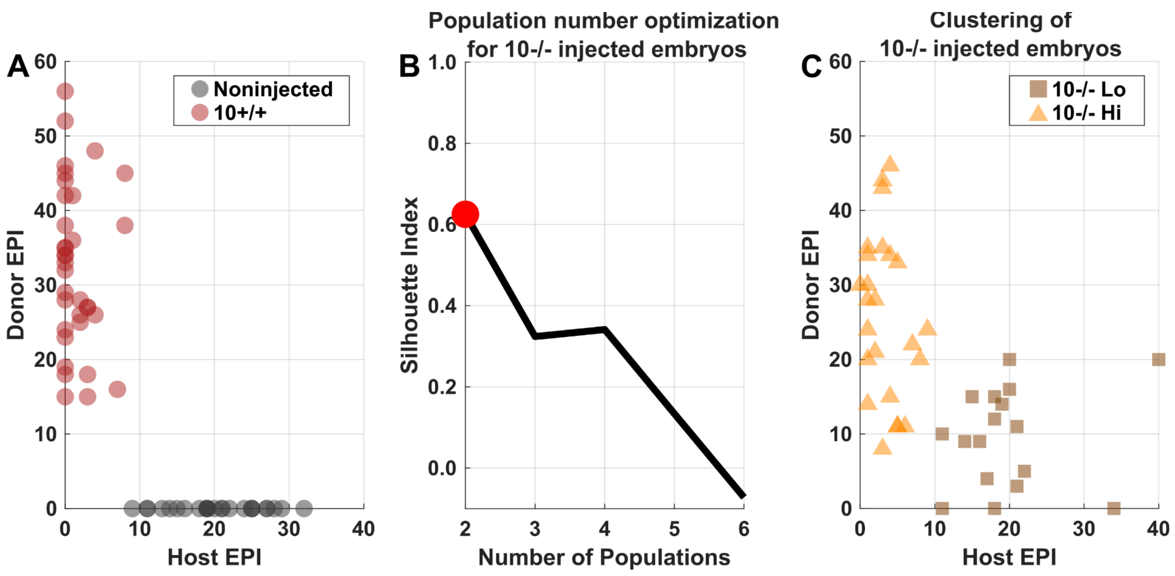

Fig. S2. k-means clustering identifies two populations in the 10 *Fgf4*$^{-/-}$ donor ESC injected embryos. A. EPI composition of noninjected (black circles) and 10 *Fgf4*$^{+/+}$ donor ESC injected (red circles) embryos. **B.** Black line: silhouette index from k-means clustering for different numbers of populations of *Fgf4*$^{+/+}$ donor ESC injected embryos; red dot: optimal number of populations. **C.** EPI composition of *Fgf4*$^{-/-}$ donor ESC injected embryos grouped by k-means clustering with optimal number of populations revealing a low (Lo) (brown squares) and high (Hi) (yellow triangles) donor cell contribution population.

Development • Supplementary information

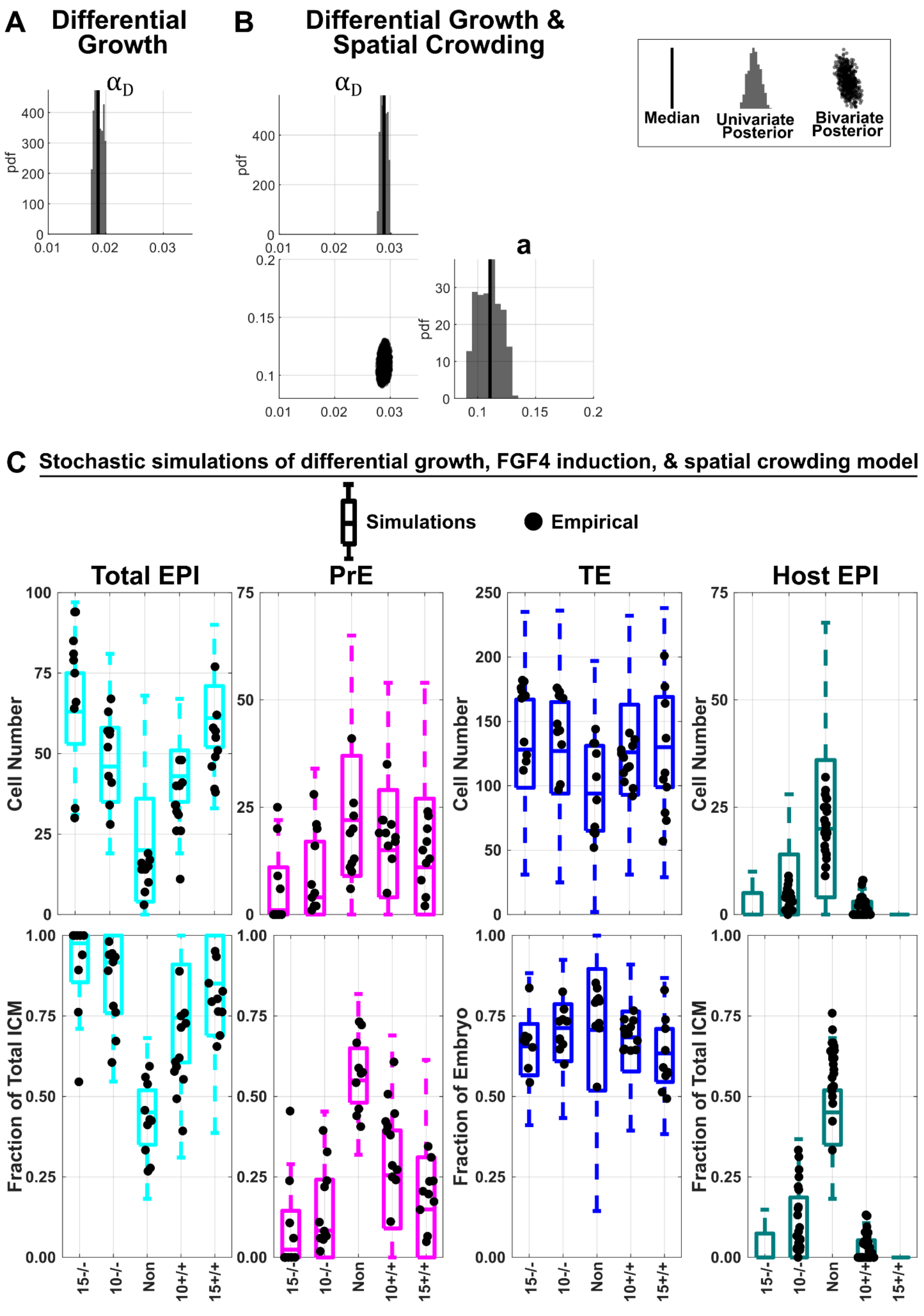

Development • Supplementary information

Fig. S3. Posterior distributions of inferred parameters for different models of chimera formation. Univariate (on diagonal histograms, vertical line shows median) and bivariate (off diagonal scatter plots) posterior distributions of inferred parameters for models with differential growth for donor cells (**A**) and with differential growth and spatial crowding (**B**). Parameters are: α_D, donor cell net growth rate; **a**, crowding factor. Not shown is the posterior for feedback parameter n which took on the value of 1 for all accepted parameter sets in the differential growth and spatial crowding model. Priors were uniform over the ranges shown in the plots. ABC thresholds (ε) were 125,000 for the model with differential growth and 62,000 for the model with differential growth and spatial crowding. **C.** Stochastic simulations of the chimera model using the Gillespie algorithm, run with the posterior median parameters from the differential growth, FGF4 induction, and spatial crowding model. Shown are 1,000 independent realizations (box plots).

Table S1. Summary of statistical analyses corresponding to Figures 2E–G and 3G–I. Summary statistics (mean, standard deviation, and N) and p-values from N-way ANOVA with Tukey's honestly significant difference (HSD) post hoc test are provided for each experimental condition. Statistical significance was assessed at $\alpha = 0.05$.

Available for download at
https://journals.biologists.com/dev/article-lookup/doi/10.1242/dev.204518#supplementary-data
