# Supplementary material for: Donor embryonic stem cells displace host cells of 8-cell-stage chimeras to the extra-embryonic lineages by spatial crowding and FGF4 signalling
Source: Development. 2025 Jun 25;152(12):dev204518. doi: 10.1242/dev.204518 (PMC12273629; doi:10.1242/dev.204518)
Supplement: Supplementary file 1 [file develop-152-204518_review_history.pdf]

Donor embryonic stem cells displace host cells of 8-cell-stage chimeras to the extra-embryonic lineages by spatial crowding and FGF4 signalling

Stanley E. Strawbridge, Anna Katharina Schrattel, Peter Humphreys, Kenneth A. Jones, Jérôme Artus, Anna-Katerina Hadjantonakis, Alexander G. Fletcher and Jennifer Nichols
DOI: 10.1242/dev.204518

Editor: James Briscoe

Review timeline
Original submission: 8 November 2024
Editorial decision: 4 December 2024
First revision received: 2 May 2025
Editorial decision: 16 May 2025
Second revision received: 20 May 2025
Accepted: 21 May 2025

Original submission

First decision letter

MS ID#: dev.204518

MS TITLE: Donor embryonic stem cells impede host epiblast specification in 8-cell stage chimeras by crowding and FGF4 signalling

AUTHORS: Stanley E. Strawbridge; Anna Katharina Schrattel; Peter Humphreys; Ken A. Jones; Jérôme Artus; Anna-Katerina Hadjantonakis; Alexander G. Fletcher; Jennifer Nichols

Dear Dr Strawbridge,

I have now received all the referees' reports on the above manuscript, and have reached a decision. The referees' comments are appended below, or you can access them online: please go to:

As you will see, the referees express considerable interest in your work, but have some significant criticisms and recommend a substantial revision of your manuscript before we can consider publication. The reviewers raise some questions about the mathematical modelling, specifically the use of deterministic continuous models for small cell populations and the interpretation of Bayesian posterior distributions. Please respond to these. In addition, the experimental data needs clarification, particularly regarding the differences in the analyses presented in Figures 2 and 3, and the rationale for subdividing the Fgf4-/- data. The points raised about the interpretation of results, and the conclusions about host cell displacement to TE lineage by Fgf4-/- ESCs, should also be addressed. Please note the referees' requests for clarifications and additional methodological details about cell counting, statistics, and transplantation protocols.

If you are able to revise the manuscript along the lines suggested, which may involve further experiments, I will be happy to receive a revised version of the manuscript. Your revised paper will be re-reviewed by one or more of the original referees, and acceptance of your manuscript will depend on your addressing satisfactorily the reviewers' major concerns. Please also note that Development will normally permit only one round of major revision. If it would be helpful, you are

© 2025. Published by The Company of Biologists under the terms of the Creative Commons Attribution License (https://creativecommons.org/licenses/by/4.0/).

welcome to contact us to discuss your revision in greater detail. Please send us a point-by-point response indicating your plans for addressing the referees' comments, and we will look over this and provide further guidance.

Please attend to all of the reviewers' comments and ensure that you clearly highlight all changes made in the revised manuscript. Please avoid using 'Tracked changes' in Word files as these are lost in PDF conversion. I should be grateful if you would also provide a point-by-point response detailing how you have dealt with the points raised by the reviewers in the 'Response to Reviewers' box. If you do not agree with any of their criticisms or suggestions please explain clearly why this is so.

Reviewer 1

SUMMARY OF THE ADVANCE MADE IN THIS PAPER AND ITS POTENTIAL SIGNIFICANCE TO THE FIELD

By combining mathematical modeling and experimental chimera formation, Strawbridge and colleagues investigate the allocation of cells to distinct lineages in mouse preimplantation embryos. Transplanted ESCs displace host cells from the epiblast, with Fgf4+/+ and Fgf4-/- ESCs showing distinct impacts on the allocation of host cells to the primitive endoderm. Based on these data, the authors propose a model that faithfully recapitulates host cell exclusion driven by physical crowding and FGF4 signaling.
The manuscript is mostly clear to experts of the field and the figures comprehensively report on the details of the experimental result. Some methodological details are missing. The conclusion on the allocation to TE specifically for Fgf4-/- transplants is not sufficiently supported by the data.

SUGGESTIONS TO AUTHORS

1. Some terms may be confusing to the readers.
The word "injection" for the addition of ESCs to morula could be misleading. We understand that ESCs injection may be standard phrasing when it comes to the insertion of ESCs inside the blastocoel but this term could lead readers to believe that ESCs are injected inside the morula (in between the host cells), which is unlikely to be the case. The words "graft" or "transplantation" would be clearer in our opinion.
During the allocation of ICM cells to Epi and PrE, there is a time window during which some cells are already biased towards Epi and PrE while other ICM cells are not. Without this clarification explicitly mentioned, we feel that sentences such as "FGF4 signalling from the ICM and epiblast" or "three cell types within the ICM" could lead readers to confuse the ICM as a structure and as a lineage. In this and similar instances, we would suggest to systematically use "unspecified ICM" (as done in the 1st paragraph) and "specified Epi".
In the first paragraph, the word "bipotent" applied solely to the ICM is confusing since the trophectoderm is also bipotent and will give rise to polar and mural TEs.
In the 2nd paragraph, "non-physiological concentrations of exogenous FGF4" are mentioned. While we agree that this statement is most likely correct, it is difficult to assert this without knowing the physiological concentrations in the first place. The current phrasing suggests that we would in fact know about the physiological concentrations. Could the authors clarify this?
For the readers to understand the results, the introduction could add more basic information regarding the facts that transplanted ESCs only contribute to Epi or that laminin is deposited between Epi and PrE for example.
The first sentence of the abstract states that TE and ICM specification occurs during mouse embryo compaction. This is incorrect. Compaction in the mouse takes place at the 8-cell stage while TE and ICM specification only begins at the 16-cell stage.
In the sentence "Building on this model……… donor ESCs can impede host cells from contributing to the epiblast, with Fgf4-/- donor ESCs yielding a smaller PrE and larger TE", the phrasing suggests that donor cells contribute to the formation of TE, which is not the case.
In the sentence "This suggests that PrE feedback is necessary to drive timely ICM specification", we think the word "necessary" is incorrect. The simulations show that PrE feedback is sufficient to explain the proportion of cell types, but there could be other explainations.

2. Some methodological details need to be explained.

© 2025. Published by The Company of Biologists under the terms of the Creative Commons Attribution License (https://creativecommons.org/licenses/by/4.0/).

While the experiments performed by the authors may appear trivial to them, more explaination on how experiments are performed should be provided for readers to understand them. How do the authors stage the morula for their transplantation experiments (strictly 8-cell stage, compacted or uncompacted)? Are ESCs deposited at the surface of the embryo or injected inside the cell cluster? Like the simulation figures, the experimental figures would strongly benefit from having a schematics of how and when the ESCs are grafted.
In Fig 2 and 3 the number of Epi and PrE cells as well as their proportions are different in the control embryos. The methods used to count cells and cell types are also different, for reasons that are not explained. Can the authors clarify this? Is the counting method at the origin of the discrepancy? The automated cell counting and allocation is well described in the method section but the manual counting and allocation is not explained. Could the authors elaborate on this? In particular, how are cells allocated to the different lineages? What are the objective criteria that would allow someone else to find the same number when looking at the raw data?
The statistics are sometimes dificult to read in some figure panels. Could the authors provide seperate tables with the actual p values (instead of asterisks)? Also the statistical analysis should be described in the method section.

3. Some results may need to be interpreted or discussed differently.
The authors conclude that only Fgf4-/- ESCs transplants would displace host cells to the TE (e.g. last sentence of the abstract). However this is not supported by the data shown in Fig 3I in which both Fgf4+/- and -/- ESCs seem to affect the total number of cells and fraction of the embryo that are TE. The authors need to amend their conclusions or provide the data that supports them.
Also, in the result paragraph dedicated to this figure, only the absolute numbers of cells are discussed but since the total cell number is different between experimental conditions, only proportions are useful. Could the authors discuss the proportions rather than the absolute values?
Fig1H: the curve implies that PrE would be specified first. Is that supported by data or the literature?
Regarding the model of Fig 1, can the authors clarify why the embryo would reach 124 cells with a doubling time of 11 h from E2.5 to E4.5?
In the sentence " Given that ESCs are significantly smaller than the blastomeres of the E2.5 8-cell stage embryo, the ESCs may sort to the interior, while blastomeres remain on the exterior", it is not clear why cell size would promote sorting. To our knowledge, there is no data supporting the influence of cell sorting by differences in cell size. Could the authors discuss cell sorting in more details? Other cell properties are more likely to affect this phenomenon than cell size (e.g. cell contractility in MaÃ®tre et al 2016 and Dumortier et al 2019, membrane fluctuations in Yanagida et al 2022).
In Fig4, the authors describe three different models. The first model includes FGF4 induction from donor cells and considers same net growth rate as host cells. The second model includes FGF4 induction and a different net growth rate, differential growth. The third model includes FGF4 induction, differential growth, and spatial crowding. Could the author comment on the specific contribution of crowding by showing simulation with crowding but without differential growth?

In the simulations and experiments, how is apoptosis taken into account? If it is not taken into account, a sentence explicitly mentioning this could be helpful to the readers.

Reviewer 2

SUMMARY OF THE ADVANCE MADE IN THIS PAPER AND ITS POTENTIAL SIGNIFICANCE TO THE FIELD

In this manuscript, the authors present an interesting and valuable study that combines experimental data with mathematical modeling, leveraging Bayesian inference to dissect and compare competing hypotheses about the mechanisms driving host cell displacement and lineage specification during mouse embryo development. The integration of biological data with mathematical approaches is well-executed and accessible, with the results generally supporting the authors' claims. A notable strength of the paper is its estimation of rates as Bayesian distributions, which could serve as a valuable resource for other researchers investigating the same system. However, the model's simplicity may limit the accuracy of the inferred rates and mechanisms, potentially impacting its applicability. Furthermore, the feedback from the model into the experiments could be more thorough, particularly in exploring the mechanisms identified by the

© 2025. Published by The Company of Biologists under the terms of the Creative Commons Attribution License (https://creativecommons.org/licenses/by/4.0/).

model. The final section, in particular, leaves many mechanistic questions unanswered, which could be addressed to strengthen the study's overall impact and significance.

SUGGESTIONS TO AUTHORS

M1.1) I found that the main limitation of the analysis is the use of deterministic continuous models in a context in which the number of cells is very small (<30). This can be seen in the data in Fig. 2D, where the embryos show a high degree of variability in tissue-specific cell number. While the ODE model chosen is a reasonable first approximation, the authors should discuss the limitations of their approximation and could consider running some Gillespie simulations with the parameters obtained to address better the heterogeneity and granularity of the data to ensure that the parameters obtained reflect the actual underlying mechanisms. This way the authors could bring strength to their claims of parameters/mechanisms reported which otherwise could just be the best fit of a insufficient model, as opposed to the identification of a good model.

M1.2) Regarding the spread of the data in comparison with the ODE results, it is unlikely that the tight posterior distributions from the Bayesian analysis reflect the actual inferred knowledge of those parameters. In other words, how believable is the spread of the posterior (credibility zones of the parameters given the data) if most of the data falls far from the simulations? Is perhaps the distance funcion used in ABC to strict? On the other hand, if the spread of the distributions is not informative, why running ABC and not a traditional point minimization?

M2.1) During the first section of the paper, the authors investigate the possibility of signalling from primitive endorderm into epiblast differentiation. This is a very interesting question, but I think that the analysis does not fully support that claim (as they will explore later in the paper). In particular the authors prove that a model in which differentiation to epiblast has a constant rate is worse at fitting the data than a model in which there is a dependence on the number of primitive endoderm cells. There are many reasons by which this could be happening:

- There could be feedback of epiblast cells into themselves
- The rates could change in time as ICM cells progress
- Simply that a model with 1 more parameter feeds in general better the data

The authors should discuss carefully why PrE feedback is necessary among other possibilities. One of the advantadges of Bayesian inference is the possibility for hypothesis testing. Perhaps they could use Bayes factor to compare them? Another possibility is to generate a larger model (as it is done later in the text) making possible to distinguish between models.

M2.2) Similarly, to be more straightfoward about the quality of the fit, it would be good to have an additional line in Fig. 2D showing the average or median of the total cell number to have a better visualization of the deviation between the model and the data.

M3) The authors mention that they explore different non-linearities by chosing discrete values of 0,1,2 for the feedback exponents. Nevertheless, there is no reason to assume that the non-linearities must take integer values. Why is this assumption necessary? In addition, if all the selected fits took a value =2, why did not the authors expand the prior i.e. try values m=3 for which the fitting might improve?

M4) The clustering of populations to differentiate Lo/Hi does not have a clear boundary that can be distinguished by eye. In addition, the silhouette analysis is not valid in this case since it can only be used to support that 2 clusters are better than >2. But it cannot be used to support than 2 clusters is better than 1 (i.e. absence of clusters). This is becuause the silhouette score requires to have more than 1 cluster to generate the metric. How necessary is this division to support the claims of the paper? In the end the mechanism for distinguising between these two populations is not explored in the manuscript.

M5) In order to include all the chimera observations in the new model the authors develop 3 extensions of the original model. In particular the flexibility of allowing different proliferation rates for donor and host cells predicts a 50% increase of proliferation rate for hosts cells. But also predicts a change in the proliferation rate of host cells with respected to the original model (rate

© 2025. Published by The Company of Biologists under the terms of the Creative Commons Attribution License (https://creativecommons.org/licenses/by/4.0/).

of 0.06 for the data in Fig. 2D). I missed an explanation for this missmatch that is required to bridge the 2 sets of data. Similarly, if we take the new parameters from the extended model and use them against the data of Fig. 2D do we recover the good fits?

M6.1) I also missed the mechanism of cell crowding. If the effect is physical (crowded cells push each other) there should be no difference between host and donor cells. Nevertheless, the crowding term only contains a dependence in the donor cell number (D^+ + D^-)

M6.2) If there is a possibility that cells proliferate at different rates, should not this be incorporated also in the initial model for the different cell types. If the model picks ups that all the host cells from all the tissues must have a consistent proliferation rate, different from the donor cells, it would provide stronger evidence towards the hypothesis.

Minor comments:

m1) The authors did not include their prior distributions. Where the priors chosen flat (uninformative) in the ranges provided in Fig. S1? If so, it should be indicated in the caption of the figure

m2) The notation of the Hi and Lo chimeras could be introduced more explicitly in the text to make it clearer.

m3) In page 6 amd -> and

m4) In page 6 MINs not introduced before. Also, segmentation details could perhaps be moved to Material and Methods?

m5) In page 6 "we say a numerical increase in PrE cell number, but this increase was not significant.". If the increase was not statistically significant, it should not be claimed that the increase was observed to start with. Specially when that trend is lost for 15+/+.

m6) Could the authors explain in more details the choice of summary statistic? In the first term the authors capture the difference in state space to remove the temporal component. If this is the case, should not the full state vector \vec X(t) and measured data \vec X^n be compared simulateneously? e.g. min_t ||\vec X(t) - \vec X^n||. With the current definition each state component (B,C,T,...) is evaluated at different timepoints.

Reviewer 3

SUMMARY OF THE ADVANCE MADE IN THIS PAPER AND ITS POTENTIAL SIGNIFICANCE TO THE FIELD

Strawbridge and colleagues use a combination of modelling and chimera experiments with wild-type and Fgf4 mutant ES cells to test if and how proportions of embryonic and extraembryonic lineages are regulated in the mouse embryo. Their initial modelling suggests a positive effect from PrE lineage for Epi specification, but this is not followed up. More significantly, their experimental data and an extended model indicate that both wild-type and Fgf4 mutant ES cells displace host cells towards the TE lineage early on, but that the subsequent displacement of host ICM cells towards the PrE lineage depends on FGF4 expression by the donor cells.

The use of Fgf4 mutant ESCs in chimera assays to probe mechanism for lineage proportioning is the main novelty of this manuscript. In doing so, the authors find that, in addition to the well-known influence of donor-derived FGF4 on the specification of host ICM cells, donor cells can also displace host cells towards the TE. This phenomenon that has received much less attention so far, and constitutes interesting and important progress that fits well in Development.

The experimental basis for the authors conclusions is somewhat thin though - they essentially only report a single type of experiment here, which has been analysed in two slightly different ways. Furthermore, I found the overall presentation of the data, particularly the differences between the experiments reported in Fig. 2 and Fig. 3 somewhat hard to follow at a first read. This is

© 2025. Published by The Company of Biologists under the terms of the Creative Commons Attribution License (https://creativecommons.org/licenses/by/4.0/).

exacerbated by the fact that some results of the two experiments do not fully align, e.g. the reduction of PrE proportions in 10+/+-injected embryos. I recommend that the authors improve on the presentation and linkage between the experiments.

Please find some specific comments below.

SUGGESTIONS TO AUTHORS

1. Fig. 1D: Can the authors quantify model performance, e.g. by calculating R2 values? Especially for the dynamics of the Epi compartment, it is not immediately clear that the model with feedback performs much better than the one without. It is also not really surprising that a more complex model fits the data better than a simpler one, since there are simply more free parameters (this applies especially to the fits in Fig. 4 D- F). Ideally, the authors should find ways to account for this increase in degree of freedom when evaluating model performance, or least discuss it as a caveat.

2. Upon a cursory read it is not immediately obvious what is the difference between the datasets described in Figs. 2 and 3. From what I understand in Fig.2, the authors focus on lineage allocation in the ICM, using the dsRed-label to distinguish between donor and host cells, while in Fig. 3 they do not have information on the origin of the cells, but analyze TE numbers in addition. I suggest to make these differences very clear - a consistent color code between the two figures, and/or clearer schemes that illustrate the difference between the experiments could help.

3. The subdivision of the 10-/- embryos in Fig. 2 is not well motivated and makes the analysis somewhat dubious. Is there any biological reason why there are two classes, e.g. do they correspond to two separate experimental sessions? Unless the authors can explain more convincingly why they chose to split the data, the current approach heavily undermines their conclusions.

4. The authors observe a reduction of the fraction of PrE cells in 10 +/+ injected embryos in Fig. 3, but not in Fig. 2. What is the casue of these discrepancies?

5. The displacement towards TE seems to be more pronounced when using Fgf4-mutant cells (Fig. 3I). Can the authors speculate why this is?

6. Fig. 4: Why were the different growth rates in the extended model estimated from the lineage proportions? Since the authors know the number of donor cells at the beginning and the end of the experiment (Fig. 2), as well as the number of total cells (donor + host, Fig. 3), I would assume that it should be possible to estimate growth rates for both donor and host cells from the cell numbers. Using such independent estimates would make this modelling part much stronger.

7. Their final conclusion (Fig. 4G) applies to an experimental setup where chimaeras are formed early, when lineage segregation between the TE and the ICM has not yet taken place. What is the authors prediction for blastocyst injection of ESCs? I believe that this would be an extremely informative experiment to further probe the proposed model. If performing the experiment is not possible, the authors might want to discuss it, and perhaps also put it in context of the literature.

Minor

8. Fig. 1D: Difference between with and w/o feedback curves are hard to see.

9. The labels in Fig. 2C, D are hard to get - what is donor, what is host?

10. The authors write: "Together this suggests that donor cells are able to
drive host cells into the TE compartment during the first cell-fate decision. In the instance where the donor cells are Fgf4-/-, host cells specify into either EPI or PrE." This is confusing - how can host cells still decide between Epi and PrE if they've been pushed to TE already?

© 2025. Published by The Company of Biologists under the terms of the Creative Commons Attribution License (https://creativecommons.org/licenses/by/4.0/).

11. I am not sure the title ideally captures the main message of the manuscript - what the donor cells do is not so much impede epiblast specification, they rather displace host cells towards extraembryonic lineages.

First revision

Author response to reviewers' comments

Detailed response to reviews

We thank the anonymous reviewers for their constructive comments. We hope that our revisions and responses address their concerns.

Reviewer 1 Comments for the Author:

The manuscript is mostly clear to experts of the field and the figures comprehensively report on the details of the experimental result. Some methodological details are missing. The conclusion on the allocation to TE specifically for Fgf4-/- transplants is not sufficiently supported by the data.

We thank the reviewer for their feedback, which we address point-by-point below.

1. Some terms may be confusing to the readers.
The word "injection" for the addition of ESCs to morula could be misleading. We understand that ESCs injection may be standard phrasing when it comes to the insertion of ESCs inside the blastocoel but this term could lead readers to believe that ESCs are injected inside the morula (in between the host cells), which is unlikely to be the case. The words "graft" or "transplantation" would be clearer in our opinion.

The term “injection” is indeed standard phrasing in the field for both blastocyst and morula stage embryos. As the reviewer notes, in the blastocyst stage the donor cells need to be inserted through the trophectoderm. In morulae, the donor cells do not need to be placed inside the embryo, since they will tend to sort to the interior because of size and cell surface differential properties. Examples of usage of “injection” include the influential papers:

- Brook & Gardner (1997). The origin and efficient derivation of embryonic stem cells in the mouse. *Proc Natl Acad Sci USA* 94:5709-12.
- Humięcka et al. (2016). ESCs injected into the 8-cell stage mouse embryo modify pattern of cleavage and cell lineage specification. *Mech Dev* 141:40-50.
- Poueymirou et al. (2007). F0 generation mice fully derived from gene-targeted embryonic stem cells allowing immediate phenotypic analyses. *Nat Biotech* 25:91-9.

We politely disagree that using alternatives such as “graft” or “transplantation” would be clearer. Indeed, they may cause unnecessary confusion among developmental biologists. We therefore respectfully propose to leave references to “injection” as they stand.

During the allocation of ICM cells to Epi and PrE, there is a time window during which some cells are already biased towards Epi and PrE while other ICM cells are not. Without this clarification explicitly mentioned, we feel that sentences such as "FGF4 signalling from the ICM and epiblast" or "three cell types within the ICM" could lead readers to confuse the ICM as a structure and as a lineage. In this and similar instances, we would suggest to systematically use "unspecified ICM" (as done in the 1st paragraph) and "specified Epi".

We apologise for this lack of clarity and have made the reviewer’s suggested change regarding “unspecified ICM”. However, we politely disagree with their suggested change regarding “specified EPI”, as this goes against standard phrasing in the field, and respectfully propose to leave references to “EPI” as they stand.

© 2025. Published by The Company of Biologists under the terms of the Creative Commons Attribution License (https://creativecommons.org/licenses/by/4.0/).

In the first paragraph, the word "bipotent" applied solely to the ICM is confusing since the trophectoderm is also bipotent and will give rise to polar and mural TEs.

Again, we politely disagree with the reviewer, in that “bipotent ICM” is standard phrasing in the field - see e.g.

- Nichols et al. (2009). Suppression of Erk signalling promotes ground state pluripotency in the mouse embryo. *Development* 136:3215-22.
- Morris et al. (2010). Origin and formation of the first two distinct cell types of the inner cell mass in the mouse embryo. *Proc Natl Acad Sci USA* 107:6364-9.
- Thompson et al. (2022). Extensive co-binding and rapid redistribution of NANOG and GATA6 during emergence of divergent lineages. *Nat Commun* 13:4257.

and does not imply anything about the potency of the trophectoderm. As such, we respectfully propose to leave this text as it stands.

In the 2nd paragraph, "non-physiological concentrations of exogenous FGF4" are mentioned. While we agree that this statement is most likely correct, it is difficult to assert this without knowing the physiological concentrations in the first place. The current phrasing suggests that we would in fact know about the physiological concentrations. Could the authors clarify this?

To address this, we have replaced the text “high, non-physiological concentrations of exogenous FGF4” with “sufficiently high concentrations of exogenous FGF4”.

For the readers to understand the results, the introduction could add more basic information regarding the facts that transplanted ESCs only contribute to Epi or that laminin is deposited between Epi and PrE for example.

To address this, we have removed the text “ESCs can be re-incorporated into […] the germ line” from the first paragraph, and amended the second paragraph to begin “The dynamic allocation of the three lineages can be perturbed in myriad ways, such as re-incorporation of ESCs into [...] These donor cells can re-enter normal development from the preimplantation EPI stage [...] Injecting donor ESCs into…”;. We added the text “Basement membrane components including [...] thus implicating PrE-produced basement membrane in EPI establishment and/or maintenance” to the end of the first paragraph.

The first sentence of the abstract states that TE and ICM specification occurs during mouse embryo compaction. This is incorrect. Compaction in the mouse takes place at the 8-cell stage while TE and ICM specification only begins at the 16-cell stage.

We thank the reviewer for noting this oversight and have amended “During” to “Following”.

In the sentence "Building on this model……… donor ESCs can impede host cells from contributing to the epiblast, with Fgf4-/- donor ESCs yielding a smaller PrE and larger TE", the phrasing suggests that donor cells contribute to the formation of TE, which is not the case.

We have clarified this ambiguity, by amending the text “with *Fgf4*$^{-/-}$ donor ESCs yielding a smaller PrE and larger TE” to “with a smaller PrE and larger TE observed in the latter case”.

In the sentence "This suggests that PrE feedback is necessary to drive timely ICM specification", we think the word "necessary" is incorrect. The simulations show that PrE feedback is sufficient to explain the proportion of cell types, but there could be other explainations.

We agree that “necessary” may be considered an over-statement, so have amended “necessary” to “sufficient”, as suggested.

2. Some methodological details need to be explained.
While the experiments performed by the authors may appear trivial to them, more explaination on how experiments are performed should be provided for readers to understand them. How do the authors stage the morula for their transplantation experiments (strictly 8-cell stage, compacted or uncompacted)? Are ESCs deposited at the surface of the embryo or injected inside

© 2025. Published by The Company of Biologists under the terms of the Creative Commons Attribution License (https://creativecommons.org/licenses/by/4.0/).

the cell cluster? Like the simulation figures, the experimental figures would strongly benefit from having a schematics of how and when the ESCs are grafted.

Morulae are generally obtained from mouse oviducts on the morning of the third day after mating. Optimally, they will be at the uncompacted 8-cell stage, enabling donor cells injected through the zona to become incorporated within the morula as it compacts. Embryo stages can vary within and between litters. Those recovered at the 4-cell stage can be cultured to the 8-cell stage. Embryos that have already compacted may be decompacted by brief culture in calcium-free medium for a few minutes before injection. Occasional abnormal embryos may be discarded, but otherwise all embryos are used. For the injection procedure, embryos and donor cells are placed in drops of medium under oil on the microscope stage of the injection rig. Embryos are immobilised by means of a suction-mediated holding pipette.
The desired number of separated ESCs are aspirated into the injection pipette. A small hole, just big enough to insert the injection pipette, is made in the zona pellucida opposite the holding pipette in a region of maximal space between blastomeres using a laser connected to the microscope's objective. The injection pipette is gently pushed through the hole in the zona and the donor cells deposited away from the hole between zona and host cells.
Embryos are then returned to appropriate culture medium and incubated until the following day. This method has been detailed in Poueymirou et al. (2006) and, to address the reviewer's point, we have now made reference to this manuscript in our *Materials and Methods* section.

In Fig 2 and 3 the number of Epi and PrE cells as well as their proportions are different in the control embryos. The methods used to count cells and cell types are also different, for reasons that are not explained. Can the authors clarify this? Is the counting method at the origin of the discrepancy?

We thank the reviewers for noting these discrepancies. While the counting methods could lead to differences in cell numbers and proportions, we believe it is more likely to be due to different operators performing the injections. The experiments in **Fig. 2** and **Fig. 3** were conducted independently, on different days, and by different operators. Specifically, the injections in **Fig. 2** were performed by a more experienced operator, while those in **Fig. 3** were performed by a trainee. We suspect that reduced cell viability in **Fig. 3** may have contributed to the lower efficiency of chimera formation, likely due to the longer injection times associated with training. This could explain both the subtle shifts in lineage proportions and the observed reduction in PrE contribution in the 10+/+ group in **Fig. 3** that is not seen in **Fig. 2**. We have included this clarification in the *Materials and Methods* section.

The automated cell counting and allocation is well described in the method section but the manual counting and allocation is not explained. Could the authors elaborate on this? In particular, how are cells allocated to the different lineages? What are the objective criteria that would allow someone else to find the same number when looking at the raw data?

We thank the reviewer for highlighting this omission. We have now updated the *Materials and Methods* section to include a detailed description of our manual cell counting approach: "Manual cell counting of ICMs from embryos and chimeras collected at E2.5+48 hrs was performed on confocal z-stacks using Fiji (ImageJ) with the 'Cell Counter' plugin. Individual cells were scored and assigned to lineages based on fluorescence marker expression:
SOX2-positive cells were classified as EPI, GATA4-positive cells as PrE, and cells double-positive for DsRed and SOX2 as donor-derived EPI."

The statistics are sometimes dificult to read in some figure panels. Could the authors provide seperate tables with the actual p values (instead of asterisks)? Also the statistical analysis should be described in the method section.

Given the volume of data, we respectfully suggest that it is not practical to include all individual p-values in the main text or figures. However, we have made the complete set of statistical analyses available at the following links:

Fig 2:
https://github.com/stanleystrawbridge/strawbridge_et_al_2024/tree/main/results/chimera_ex

© 2025. Published by The Company of Biologists under the terms of the Creative Commons Attribution License (https://creativecommons.org/licenses/by/4.0/).

clusion/stats

Fig 3:
https://github.com/stanleystrawbridge/strawbridge_et_al_2024/tree/main/results/chimera_crowding/stats

We have amended the *Data Availability* statement to reflect this. Regarding description of our statistical analysis, please note that in the captions to **Fig. 2** and **Fig. 3**, we already state: "Pairwise comparisons were carried out by N-way ANOVA, p-values: *, $0.05 \geq p > 0.01$; **, $0.01 \geq p > 0.001$; ***, $0.001 \geq p > 0.0001$; ****, $0.0001 \geq p$". N-way ANOVA is a standard statistical analysis method; full details can be found in our code at:
https://github.com/stanleystrawbridge/strawbridge_et_al_2024/blob/main/funs/model_inference.m

3. Some results may need to be interpreted or discussed differently.
The authors conclude that only Fgf4-/- ESCs transplants would displace host cells to the TE (e.g. last sentence of the abstract). However this is not supported by the data shown in Fig 3I in which both Fgf4+/- and -/- ESCs seem to affect the total number of cells and fraction of the embryo that are TE. The authors need to amend their conclusions or provide the data that supports them.

As indicated in **Fig. 3I**, we found a statistically significant difference in TE number between 15-/- and non-injected ($p < 0.001$), and between 10-/- and non-injected ($p < 0.01$); but we found no statistically significant difference between 15+/+ and non-injected, nor between 10+/+ and non-injected. Thus, we politely disagree with the reviewer's statement that both Fgf4+/+ and -/- ESCs seem to affect the total number of cells of the embryo that are TE. As the reviewer notes, both Fgf4+/+ and -/- ESCs seem to affect the proportion of cells in the embryo that are TE. However, this is due to the increased size of the total EPI, including donor cells (**Fig. 3G**, Cell Number). We now clarify this point when discussing **Fig. 3I** in the main text: "The fractions of 15+/+ and 15-/- chimeras composed of TE cells were significantly smaller than non-injected embryos, which can be attributed to the corresponding increase in EPI cell number in these chimeras."

Also, in the result paragraph dedicated to this figure, only the absolute numbers of cells are discussed but since the total cell number is different between experimental conditions, only proportions are useful. Could the authors discuss the proportions rather than the absolute values?

Please note that we already discuss EPI and PrE proportions in this paragraph: "Consistent with the previous experiment, we observed a larger EPI in all chimera conditions in both cell number <u>and fraction</u> of the ICM, as compared to non-injected embryos (**Fig. 3G**) [...] Moreover, we saw a reduction in PrE cell number <u>and ICM fraction</u> for 10-/- and 15-/- chimeras (**Fig. 3H**) [...] As compared to the previous 10+/+ chimeras, we saw a decrease in <u>the PrE fraction of the ICM</u> in 10+/+ chimeras". In response to the previous comment, we now also discuss the embryo fraction composed of TE cells.

Fig1H: the curve implies that PrE would be specified first. Is that supported by data or the literature?

Due to the fact that ICM is both generating and responding to FGF4, we believe that it is most likely that PrE will specify earlier than EPI. Indeed, the data from Saiz et al. 2016 show a high number of PrE cells earlier than EPI in earlier embryos. However, Plusa et al. 2008 and Grabarek et al. 2012 show that the plasticity of the EPI is lost prior to the PrE. We have added this point in the relevant part of the *Results* section.

Regarding the model of Fig 1, can the authors clarify why the embryo would reach 124 cells with a doubling time of 11 h from E2.5 to E4.5?

We thank the reviewer for catching this error. The correct value is 128 cells, and this has been corrected in the main text. This estimate is based on an average doubling time of 11 hours, during which the embryo undergoes approximately four rounds of division from 8 to 16, 32, 64, and finally 128 cells between E2.5 and E4.5.

© 2025. Published by The Company of Biologists under the terms of the Creative Commons Attribution License (https://creativecommons.org/licenses/by/4.0/).

In the sentence " Given that ESCs are significantly smaller than the blastomeres of the E2.5 8-cell stage embryo, the ESCs may sort to the interior, while blastomeres remain on the exterior", it is not clear why cell size would promote sorting. To our knowledge, there is no data supporting the influence of cell sorting by differences in cell size. Could the authors discuss cell sorting in more details? Other cell properties are more likely to affect this phenomenon than cell size (e.g. cell contractility in Maître et al 2016 and Dumortier et al 2019, membrane fluctuations in Yanagida et al 2022).

We apologise for the lack of clarity here. We were referring to the process of granular convection or the so-called "brazil nut effect", whereby differently sized 'particles' in granular materials sort themselves into distinct layers when shaken, vibrated or sheared (see e.g. Gajjar et al. (2021). Size segregation of irregular granular materials captured by time-resolved 3D imaging. *Sci Rep* 11:8352). Intriguingly, a similar effect was recently suggested to play a role in EPI/PrE sorting, with asymmetric oscillation of the blastocyst cavity facilitating EPI/PrE segregation by enhancing cell-cell contact fluctuations within the ICM and initiating convergent cell flows (Guo et al. (2025). Cavity oscillation drives pattern formation in early mammalian embryos. *Cell Rep* 44:115342). Regardless, to address the reviewer's comment, we have amended this sentence in the text to: "It has been shown that the ESCs sort to the interior, while blastomeres remain on the exterior (Humięcka et al. 2016). The mechanisms underlying this process are not yet established, but could include differential cell contractility (Maître et al. 2016) or membrane fluctuations (Yanagida et al. 2022)."

In Fig4, the authors describe three different models. The first model includes FGF4 induction from donor cells and considers same net growth rate as host cells. The second model includes FGF4 induction and a different net growth rate, differential growth. The third model includes FGF4 induction, differential growth, and spatial crowding. Could the author comment on the specific contribution of crowding by showing simulation with crowding but without differential growth?

We thank the reviewer for this insightful suggestion. In response, we implemented a fourth model incorporating FGF4 induction and spatial crowding, but without differential growth. Specifically, we used the posterior median parameters from the embryo model with PrE feedback, combined with the chimera model parameters that included FGF4 induction and crowding, while fixing donor proliferation rates to match those of the host. As shown by the red dash-dotted line in the figure below, this model predicts an unrealistically large epiblast compartment, particularly in the 15+/+ and 15-/- conditions. These predictions diverge significantly from the empirical data, suggesting that spatial crowding alone, without differential proliferation, fails to explain the observed lineage distributions. Therefore, we conclude that both differential growth and crowding are necessary to accurately capture the dynamics of chimera development.

Although this exercise provided useful insights, we have opted not to include it in the manuscript to maintain clarity and narrative coherence. Our initial modelling efforts, using equal proliferation rates, revealed a dramatic overgrowth of the epiblast compartment (as indicated by the light gray dotted line in the figure), which was inconsistent with experimental observations. This result highlighted the need to address differential growth early in the model development. Reintroducing a model without differential growth at a later stage, after it has already been corrected, would disrupt the logical flow of our model refinement.
Including it could confuse the reader by revisiting an already-resolved limitation without contributing new mechanistic insight. For these reasons, we present this result here in the response for completeness, but reserve the main manuscript for models that build upon each other in a constructive manner.

© 2025. Published by The Company of Biologists under the terms of the Creative Commons Attribution License (https://creativecommons.org/licenses/by/4.0/).

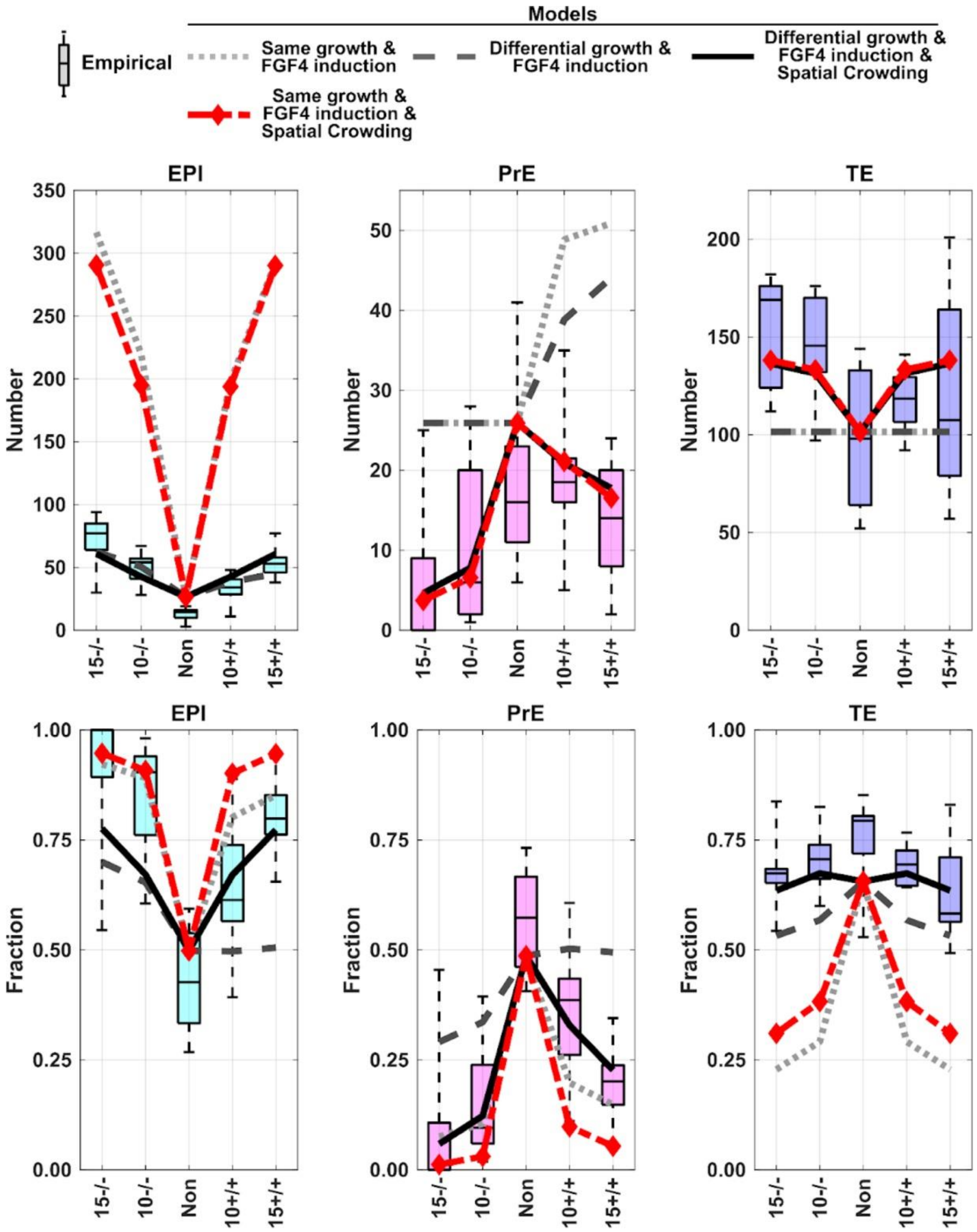

In the simulations and experiments, how is apoptosis taken into account? If it is not taken into account, a sentence explicitly mentioning this could be helpful to the readers.

As indicated in **Fig. 1C**, our model accounts for cell death via our net growth rate, α. To make this clearer, the main text “Finally, all cells proliferate with the same net growth rate α” has been amended to include “which captures both cell division and death”.

Reviewer 2 Comments for the Author:

A notable strength of the paper is its estimation of rates as Bayesian distributions, which could serve as a valuable resource for other researchers investigating the same system. However, the model's simplicity may limit the accuracy of the inferred rates and, potentially impacting its applicability. Furthermore, the feedback from the model into the experiments could be more thorough, particularly in exploring the mechanisms identified by the model. The final section, in

© 2025. Published by The Company of Biologists under the terms of the Creative Commons Attribution License (https://creativecommons.org/licenses/by/4.0/).

particular, leaves many mechanistic questions unanswered, which could be addressed to strengthen the study's overall impact and significance.

We thank the reviewer for their feedback, which we address point-by-point below.

M1.1) I found that the main limitation of the analysis is the use of deterministic continuous models in a context in which the number of cells is very small (<30). This can be seen in the data in Fig. 2D, where the embryos show a high degree of variability in tissue-specific cell number. While the ODE model chosen is a reasonable first approximation, the authors should discuss the limitations of their approximation and could consider running some Gillespie simulations with the parameters obtained to address better the heterogeneity and granularity of the data to ensure that the parameters obtained reflect the actual underlying mechanisms. This way the authors could bring strength to their claims of parameters/mechanisms reported which otherwise could just be the best fit of a insufficient model, as opposed to the identification of a good model.

We thank the reviewer for this valuable suggestion. We agree that stochastic effects can play a significant role when modelling developmental systems involving small cell numbers, such as those shown in **Fig. 2D**. While our original ODE model served as a useful first approximation, we have now extended our analysis by performing Gillespie simulations using the posterior median parameter values obtained from our ABC inference. This allows us to better capture the variability observed in the experimental data and assess the robustness of our parameter estimates under stochastic dynamics.

The results of these simulations are now included as **Fig. S1C** for the embryo model and **Fig. S3C** for the chimera model. We have also added a discussion of the implications of this stochastic analysis to the main text. In summary, our stochastic simulations are in good agreement with the empirical data.

"To better reflect the inherent randomness in systems with small cell numbers, we ran stochastic simulations using the Gillespie algorithm and the median posterior parameter values from our PrE feedback model (**Fig. S1C**). These simulations reproduced cell type distributions similar to those observed experimentally, supporting the reliability of our parameter estimates and showing that the deterministic model provides a sufficient approximation of the underlying dynamics."

"To further assess how well the GFC model reflects biological variability in small cell populations, we performed stochastic simulations using the median posterior parameter values (**Fig. S3C**). These simulations reproduced both the range and variability of lineage contributions seen in the experimental data, suggesting that the model captures key sources of noise and heterogeneity in chimera development."

M1.2) Regarding the spread of the data in comparison with the ODE results, it is unlikely that the tight posterior distributions from the Bayesian analysis reflect the actual inferred knowledge of those parameters. In other words, how believable is the spread of the posterior (credibility zones of the parameters given the data) if most of the data falls far from the simulations? Is perhaps the distance funcion used in ABC to strict? On the other hand, if the spread of the distributions is not informative, why running ABC and not a traditional point minimization?

We appreciate the reviewer's thoughtful critique. To explore this further, we repeated the ABC procedure with different tolerances. As presented in **Fig. S1B**, when we used a relatively small tolerance (ε = 13,500; acceptance rate = 0.08%), we obtained tight posteriors with median values [α, β, ρ, ζ, η, l, m] = [0.06, 0.2, 0.3, 0.0007, 0.0001, 2.0, 2.0] (1 sf).

When we instead use a larger tolerance (ε = 14,000; acceptance rate = 2%), we obtained wider posteriors as shown in the figure below, with median values [α, β, ρ, ζ, η, l, m] = [0.06, 0.6, 0.3, 0.0007, 0.0001, 2.0, 2.0] (1 sf).

© 2025. Published by The Company of Biologists under the terms of the Creative Commons Attribution License (https://creativecommons.org/licenses/by/4.0/).

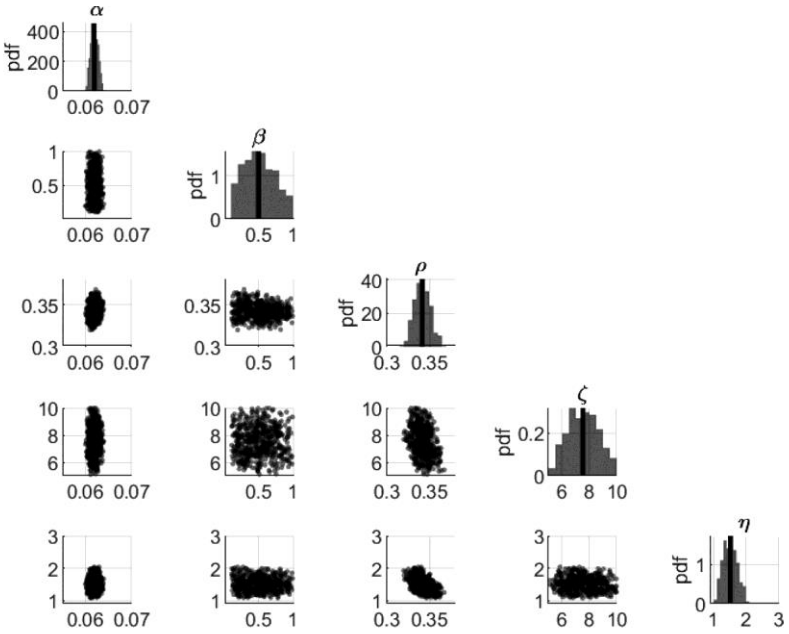

These results suggest that a traditional point estimation approach would be feasible. We agree that, in this case, the posterior distributions obtained via ABC appear tightly concentrated, and the accepted parameter sets converge toward specific values. This reflects the fact that, for our chosen distance metric and threshold, the model is highly constrained by the data and behaves similarly to a point estimation approach. However, we chose ABC precisely because we did not know *a priori* whether the posterior distributions would be informative or whether they would collapse to point estimates.

M2.1) During the first section of the paper, the authors investigate the possibility of signalling from primitive endorderm into epiblast differentiation. This is a very interesting question, but I think that the analysis does not fully support that claim (as they will explore later in the paper). In particular the authors prove that a model in which differentiation to epiblast has a constant rate is worse at fitting the data than a model in which there is a dependence on the number of primitive endoderm cells. There are many reasons by which this could be happening:

- *There could be feedback of epiblast cells into themselves*
- *The rates could change in time as ICM cells progress*
- *Simply that a model with 1 more parameter feeds in general better the data*

The authors should discuss carefully why PrE feedback is necessary among other possibilities. One of the advantadges of Bayesian inference is the possibility for hypothesis testing. Perhaps they could use Bayes factor to compare them? Another possibility is to generate a larger model (as it is done later in the text) making possible to distinguish between models.

In response to a comment by Reviewer 1, we now provide additional discussion in the Introduction of evidence for PrE signalling acting on EPI differentiation: “Basement membrane components including Laminin 511 increase the efficiency of ESC capture (Boroviak et al. 2014), and PrE is known to produce basement membrane components like Collagen 4a1 and Laminin 1 (Kang, Garg, and Hadjantonakis 2017), thus implicating PrE-produced basement membrane in EPI establishment and/or maintenance.”

We hope that this clarifies our motivation for studying this potential mechanism over the alternatives suggested by this reviewer (there could be feedback of epiblast cells into themselves; the rates could change in time as ICM cells progress), for which we are unaware of any supporting evidence. To address the reviewer’s point, we have added the following text to our concluding discussion: “While we favour the interpretation that induction of ICM to EPI is mediated by basement membrane components produced by the PrE, we acknowledge that this remains a hypothesis. Further experimental and theoretical investigation will be required to definitively resolve the underlying mechanism.”

© 2025. Published by The Company of Biologists under the terms of the Creative Commons Attribution License (https://creativecommons.org/licenses/by/4.0/).

Regarding the reviewer's suggestion that "a model with 1 more parameter feeds in general better the data", please note that in response to a comment by Reviewer 3, we now calculate Bayes factors to more systematically assess model performance to show that they PrE feedback model outperforms the no PrE feedback model.

M2.2) Similarly, to be more straightfoward about the quality of the fit, it would be good to have an additional line in Fig. 2D showing the average or median of the total cell number to have a better visualization of the deviation between the model and the data.

We thank the reviewer for this helpful suggestion. We have now added lines indicating the median total cell number to **Fig. 1D** and **Fig. S1D** to aid visual comparison between the model predictions and the empirical data.

M3) The authors mention that they explore different non-linearities by chosing discrete values of 0,1,2 for the feedback exponents. Nevertheless, there is no reason to assume that the non-linearities must take integer values. Why is this assumption necessary? In addition, if all the selected fits took a value =2, why did not the authors expand the prior i.e. try values m=3 for which the fitting might improve?

We thank the reviewer for this thoughtful question. Our aim in exploring values of 0, 1, and 2 for the feedback exponent was to distinguish between three qualitatively distinct regimes: no feedback (0), linear feedback (1), and nonlinear feedback (2). This allowed us to determine whether introducing feedback at all, and whether making it nonlinear, improved the model's ability to explain the data. We did not focus on identifying the precise degree of nonlinearity, as this was not our primary interest and is unlikely to be biologically meaningful given the limited resolution of the data. While the posterior mode indeed selected $m = 2$, we do not interpret this as evidence that $m = 2$ is optimal in a quantitative sense. Rather, it indicates that nonlinear feedback provides a better fit than either no or linear feedback. We clarify this point in the revised manuscript through the amended sentence: "To explore sufficiency of no, linear, or nonlinear feedback, parameters l and m were allowed to vary between 0, 1, and 2."

M4) The clustering of populations to differentiate Lo/Hi does not have a clear boundary that can be distinguished by eye. In addition, the silhouette analysis is not valid in this case since it can only be used to support that 2 clusters are better than >2. But it cannot be used to support than 2 clusters is better than 1 (i.e. absence of clusters). This is becuause the silhouette score requires to have more than 1 cluster to generate the metric. How necessary is this division to support the claims of the paper? In the end the mechanism for distinguising between these two populations is not explored in the manuscript.

According to Kaufman & Rousseeuw (Finding Groups in Data: An Introduction to Cluster Analysis, Wiley, New York, 1990), silhouette values in the range of 0.51–0.70 indicate a reasonable clustering structure, whereas values below 0.25 suggest that no substantial structure is present in the data. Our silhouette scores were consistently above 0.5, supporting the presence of moderate structure consistent with two biologically distinct subpopulations. We have clarified this in the main text.

Notably, this distinction is more apparent in one-dimensional histograms than in silhouette or multidimensional clustering plots. As shown in the figure below, the $10^{+}/^{+}$ embryos display a relatively unimodal distribution of host epiblast cell numbers, while the $10^{-}/^{-}$ group shows a clearly bimodal pattern, suggesting the presence of both low and high host EPI contributors.

© 2025. Published by The Company of Biologists under the terms of the Creative Commons Attribution License (https://creativecommons.org/licenses/by/4.0/).

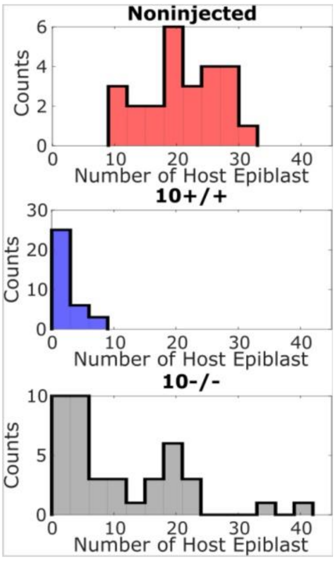

M5) In order to include all the chimera observations in the new model the authors develop 3 extensions of the original model. In particular the flexibility of allowing different proliferation rates for donor and host cells predicts a 50% increase of proliferation rate for hosts cells. But also predicts a change in the proliferation rate of host cells with respected to the original model (rate of 0.06 for the data in Fig. 2D). I missed an explanation for this missmatch that is required to bridge the 2 sets of data. Similarly, if we take the new parameters from the extended model and use them against the data of Fig. 2D do we recover the good fits?

Please note that we did not allow the host cell proliferation rate to vary in the chimera model. Instead, we fixed the host cell proliferation rate to be the posterior median value inferred from the embryo-only model (α = 0.06 h^{-1}), and allowed only the donor cell proliferation rate(α_D) to vary during the chimera model parameter inference. To clarify this, we have amended a sentence in the relevant results section to the following: "These models were simulated using the posterior median values for parameters from the base model of embryogenesis, including the host cell net growth rate."

Notably, we can obtain an independent estimate for the donor cell net growth rate for comparison with our posterior median of α_D (0.02 h^{-1}). In particular, Alexandrova et al. (Selection and dynamics of embryonic stem cell integration into early mouse embryos, *Development*, 2016) injected 5 ESCs into 8-cell stage embryos and quantified the number of deaths, divisions, and viable cells generated over a 48 h period. Approximately 12 cells were accumulated in total. Assuming exponential growth, we can therefore estimate the net growth rate via N(t) = N_0 exp(a * t) as a = (1 / t) log(N(t) / N_0) = 1/48 * log(12/5) = 0.0182 h^{-1}, in good agreement with our posterior median. We have now included the following sentence in the main text, "The inferred donor growth rate (α_D) was approximately 0.02 h^{-1}, consistent with empirical observations of ~0.018 h^{-1} based on live-cell tracking of cells derived from donor ESCs in chimeric embryos (Alexandrova et al. 2016)".

M6.1) I also missed the mechanism of cell crowding. If the effect is physical (crowded cells push each other) there should be no difference between host and donor cells. Nevertheless, the crowding term only contains a dependence in the donor cell number (D^+ + D^-)

We thank the reviewer for this thoughtful observation. The crowding term in our model is motivated by experimental findings from Humięcka et al. (2016), which demonstrate that ESCs injected into 8-cell stage embryos tend to localize to the interior, while host blastomeres exhibit increased polarization — a feature associated with TE fate. These observations suggest that donor cells may bias host cells toward the outer compartment, thereby influencing their lineage allocation. While the precise biophysical mechanism remains unclear, we have now clarified this rationale in the manuscript text where the crowding term is introduced: "Injecting donor ESCs into 8-cell embryos at embryonic day (E) 2.5 can increase TE cell numbers by moving to the interior of

© 2025. Published by The Company of Biologists under the terms of the Creative Commons Attribution License (https://creativecommons.org/licenses/by/4.0/).

the embryo and driving host blastomeres to an outer position (**Fig. 1B**) (Humięcka et al. 2016).”

M6.2) If there is a possibility that cells proliferate at different rates, should not this be incorporated also in the initial model for the different cell types. If the model picks ups that all the host cells from all the tissues must have a consistent proliferation rate, different from the donor cells, it would provide stronger evidence towards the hypothesis.

We take the reviewer’s point, and have chosen to assume the same net growth rate for all host cells for the sake of simplicity. However, partial evidence in support of this simplifying assumption is provided by a good linear fit between TE and non-TE cell numbers in our data, which we now provide as a new panel in **Fig. S1**, and refer to in the main text as follows: “Finally, for simplicity we assume that all cells proliferate with the same net growth rate α, which captures both cell division and death (**Fig. 1C**). Partial evidence in support of this simplifying assumption is provided by a good linear fit between TE and non-TE cell numbers in our data (**Fig. S1A**)”.

We have also included the following text in *Materials and Methods*:
“This simplifying assumption is supported by a strong linear relationship between TE and ICM-derived cell numbers (unspecified ICM, PrE, and EPI), with a standardized effect size of $\beta = 1.1143$, suggesting that these lineages expand at comparable rates during this developmental window (**Fig. S1A**).”

m1) The authors did not include their prior distributions. Where the priors chosen flat (uninformative) in the ranges provided in Fig. S1? If so, it should be indicated in the caption of the figure

We thank the reviewer for this helpful suggestion. We confirm that all priors were uniform over the parameter ranges shown in **Fig. S1** and **Fig. S3**, and we have now indicated this explicitly in their captions.

m2) The notation of the Hi and Lo chimeras could be introduced more explicitly in the text to make it clearer.

Please note that we already introduce this notation explicitly in the text, near the bottom of page 5: “Two groups with varying levels of chimerism were observed in the 10 *Fgf4*$^{-/-}$ ESC injected (10-/-) embryos [...] The two resulting groups were chimeras with high (Hi) and low (Lo) levels of donor cell contribution to the total EPI”.

m3) In page 6 amd -> and

We have now corrected this typo.

m4) In page 6 MINs not introduced before. Also, segmentation details could perhaps be moved to Material and Methods?

In line with the reviewer’s suggestion, we have replaced the relevant text with “Cells were segmented and cell types determined as described in *Materials and Methods*”.

m5) In page 6 "we say a numerical increase in PrE cell number, but this increase was not significant.". If the increase was not statistically significant, it should not be claimed that the increase was observed to start with. Specially when that trend is lost for 15+/+.

We have addressed this by removing the sentence “In 10+/+ chimeras, we saw a numerical increase in PrE cell number, but this increase was not significant”.

m6) Could the authors explain in more details the choice of summary statistic? In the first term the authors capture the difference in state space to remove the temporal component. If this is the case, should not the full state vector \vec X(t) and measured data \vec X^n be compared simulateneously? e.g. min_t ||\vec X(t) - \vec X^n||. With the current definition each state component (B,C,T,...) is evaluated at different timepoints.

© 2025. Published by The Company of Biologists under the terms of the Creative Commons Attribution License (https://creativecommons.org/licenses/by/4.0/).

We thank the reviewer for spotting this typo. We do indeed use min_t ||\vec X(t) - \vec X^n|| as the reviewer suggests, and have fixed the expression in the revised manuscript, which was also missing an additional penalty capturing our wish to avoid having any blastomeres or unspecified ICM cells present at our final time point in the model solution.

Reviewer 3 Comments for the Author:

Strawbridge and colleagues use a combination of modelling and chimera experiments with wild-type and Fgf4 mutant ES cells to test if and how proportions of embryonic and extraembryonic lineages are regulated in the mouse embryo. Their initial modelling suggests a positive effect from PrE lineage for Epi specification, but this is not followed up. More significantly, their experimental data and an extended model indicate that both wild-type and Fgf4 mutant ES cells displace host cells towards the TE lineage early on, but that the subsequent displacement of host ICM cells towards the PrE lineage depends on FGF4 expression by the donor cells.

The use of Fgf4 mutant ESCs in chimera assays to probe mechanism for lineage proportioning is the main novelty of this manuscript. In doing so, the authors find that, in addition to the well-known influence of donor-derived FGF4 on the specification of host ICM cells, donor cells can also displace host cells towards the TE. This phenomenon that has received much less attention so far, and constitutes interesting and important progress that fits well in Development.

The experimental basis for the authors conclusions is somewhat thin though - they essentially only report a single type of experiment here, which has been analysed in two slightly different ways. Furthermore, I found the overall presentation of the data, particularly the differences between the experiments reported in Fig. 2 and Fig. 3 somewhat hard to follow at a first read. This is exacerbated by the fact that some results of the two experiments do not fully align, e.g. the reduction of PrE proportions in 10+/+-injected embryos. I recommend that the authors improve on the presentation and linkage between the experiments.

We thank the reviewer for their feedback, which we address point-by-point below.

1. Fig. 1D: Can the authors quantify model performance, e.g. by calculating R2 values? Especially for the dynamics of the Epi compartment, it is not immediately clear that the model with feedback performs much better than the one without. It is also not really surprising that a more complex model fits the data better than a simpler one, since there are simply more free parameters (this applies especially to the fits in Fig. 4 D- F). Ideally, the authors should find ways to account for this increase in degree of freedom when evaluating model performance, or least discuss it as a caveat.

We thank the reviewer for this insightful comment. As suggested, we now include a quantitative comparison of model performance using Bayes factors, which are particularly well suited to model selection in the context of ABC, as they account for differences in model complexity and parameter dimensionality without relying on ad hoc penalization strategies like AIC or BIC. Unlike R^2 values, whose interpretation can be limited or misleading for nonlinear models such as ours, Bayes factors provide a principled approach to evaluating relative model evidence.

For the embryo model, we compared Model 1 (no feedback) and Model 2 (with PrE feedback) using the same acceptance threshold (ε = 17,300). Model 1 accepted 500 samples from 105,027 simulations, while Model 2 required only 6,289 simulations to reach the same number of accepted samples. This yields a Bayes factor (BF_{21}) of: BF_{21} = (500 / 6,289) / (500 / 105,027) = 17, indicating strong support for Model 2 over Model 1.

For the chimera simulations, we compared Model 2 (with differential growth and FGF4 feedback) and Model 3 (which also includes crowding). Using ε = 117,000, Model 2 required 26,575 simulations to obtain 500 accepted parameter sets, while Model 3 required only 585. This results in a Bayes factor (BF_{32}) of:

Hence BF_{32} = (500 / 26,575) / (500 / 585) = 45.5832, which constitutes very strong evidence in favour of Model 3.

© 2025. Published by The Company of Biologists under the terms of the Creative Commons Attribution License (https://creativecommons.org/licenses/by/4.0/).

These Bayes factors were computed following the methodology described by Toni et al. (2009) [DOI: 10.1098/rsif.2008.0172], and we now include this analysis in the main text.

These conclusions are further supported by stochastic simulations using the Gillespie algorithm (**Figs. S1C, S3C**), which recapitulate the observed variability in the experimental data and reinforce the robustness of our parameter inference under noise. This additional analysis is discussed in detail in response to Reviewer 2's comment M1.1.

2. Upon a cursory read it is not immediately obvious what is the difference between the datasets described in Figs. 2 and 3. From what I understand in Fig.2, the authors focus on lineage allocation in the ICM, using the dsRed-label to distinguish between donor and host cells, while in Fig. 3 they do not have information on the origin of the cells, but analyze TE numbers in addition. I suggest to make these differences very clear - a consistent color code between the two figures, and/or clearer schemes that illustrate the difference between the experiments could help.

We thank the reviewer for highlighting the potential confusion between the datasets in **Fig. 2** and **Fig. 3**. To clarify, **Fig. 2** focuses on lineage allocation within the ICM and distinguishes donor versus host cells using a DsRed reporter. In contrast, **Fig. 3** assesses total cell numbers across the entire embryo, including the TE compartment, but does not distinguish the origin of cells. To make this distinction clearer, we have updated the schematic in panel A of each figure to more explicitly illustrate the experimental design, including the presence or absence of donor cell labeling and the specific compartments analyzed. Please note that we have used a consistent colour scheme throughout the manuscript: EPI is shown in cyan, host EPI in dark green, donor EPI in grey, PrE in magenta, and TE in blue. We hope these clarifications and figure updates improve the readability and interpretation of the data.

3. The subdivision of the 10-/- embryos in Fig. 2 is not well motivated and makes the analysis somewhat dubious. Is there any biological reason why there are two classes, e.g. do they correspond to two separate experimental sessions? Unless the authors can explain more convincingly why they chose to split the data, the current approach heavily undermines their conclusions.

The subdivision of the $10^{-}/^{-}$ embryos was not pre-specified based on experimental batch or handling, but rather emerged from an unbiased data-driven clustering of the host epiblast cell counts. As noted in our response above to Reviewer 2's comment M4, the bimodal distribution of these counts in the $10^{-}/^{-}$ condition is clearly visible in one-dimensional histograms, in contrast to the unimodal distribution observed in the $10^{+}/^{+}$ controls. This motivated our use of unsupervised k-means clustering to identify two distinct subpopulations.

While we cannot definitively assign a biological cause in the absence of live-cell tracking, we speculate that embryos in the "Hi" subpopulation may have received more viable donor cells, while those in the "Lo" group may have experienced greater donor cell death or failed integration during the 48-hour window.

4. The authors observe a reduction of the fraction of PrE cells in 10 +/+ injected embryos in Fig. 3, but not in Fig. 2. What is the casue of these discrepancies?

As indicated in our response to a similar query from Reviewer 1, we believe that these discrepancies are most likely to be due to different operators performing the injections. The experiments in **Fig. 2** and **Fig. 3** were conducted independently, on different days, and by different operators. Specifically, the injections in **Fig. 2** were performed by a more experienced operator, while those in **Fig. 3** were performed by a trainee. We suspect that reduced cell viability in **Fig. 3** may have contributed to the lower efficiency of chimera formation, likely due to the longer injection times associated with training. This could explain both the subtle shifts in lineage proportions and the observed reduction in PrE contribution in the 10+/+ group in **Fig. 3** that is not seen in **Fig. 2**.

5. The displacement towards TE seems to be more pronounced when using Fgf4-mutant cells (Fig. 3I). Can the authors speculate why this is?

© 2025. Published by The Company of Biologists under the terms of the Creative Commons Attribution License (https://creativecommons.org/licenses/by/4.0/).

The reviewer raises an interesting observation. One possibility is that some host cells in the early embryo are responsive to FGF4 signalling, leading to their precocious specification to PrE in the case of both *Fgf4*$^{+/+}$ donor cells. These host cells may instead be displaced only into the TE in the case of *Fgf4*$^{-/-}$ donor cells. To explore this further would require additional live-imaging assays using a PrE marker like PDGFRA, and TE marker like CDX2, but these mouse lines do not exist in combination without phenotype.

6. Fig. 4: Why were the different growth rates in the extended model estimated from the lineage proportions? Since the authors know the number of donor cells at the beginning and the end of the experiment (Fig. 2), as well as the number of total cells (donor + host, Fig. 3), I would assume that it should be possible to estimate growth rates for both donor and host cells from the cell numbers. Using such independent estimates would make this modelling part much stronger.

Whilst we chose to fit parameters in the extended model using the same ABC-based approach as for the first model, we take the reviewer's point here. To address this, we can obtain an independent estimate for the growth rates of host ($H = B + T + C + E + P$) and donor ($D^{\pm}$) cells, by assuming exponential growth of these two compartments and fitting exponential functions (t) $=$ $(0)^{\alpha}{}_{t}$ and $D(t) = D(0)e^{\alpha}{}_{D}{}^{t}$ to the following estimated averages:

Cell type	Time (h)	Number	Reference
Donor	0	10	Number injected
Donor	48	30	**Fig 2E,G**; difference between total EPI and host EPI for 10-/- Hi (similar value if instead using 10+/+)
Host	0	8	Number of cells in 8-cell stage embryo
Host	48	128	Estimated from **Fig 3D**; difference between total cell number of 10-/- and donor cell number as above (similar value if instead using 10+/+)

This yields the estimates $\alpha = 0.06$ h^{-1} and $\alpha = 0.02$ h^{-1}, respectively. These estimates are in good agreement with our posterior means for these growth rates, as indicated in **Fig S1** and **Fig S3**, respectively, confirming the validity of our approach.

Additionally, please see our response to Reviewer 2's comment M4 regarding donor cell proliferation rate, as well as the newly introduced text in the main manuscript clarifying this point: "The inferred donor growth rate (α) was approximately 0.02 h^{-1}, consistent with empirical observations of ~0.018 h^{-1} based on live-cell tracking of cells derived from donor ESCs in chimeric embryos (Alexandrova et al. 2016)".

7. Their final conclusion (Fig. 4G) applies to an experimental setup where chimaeras are formed early, when lineage segregation between the TE and the ICM has not yet taken place. What is the authors prediction for blastocyst injection of ESCs? I believe that this would be an extremely informative experiment to further probe the proposed model. If performing the experiment is not possible, the authors might want to discuss it, and perhaps also put it in context of the literature.

The reviewer raises an interesting question, which has already been addressed in the literature: Poueymirou et al. (2007) showed that ESC injections at the blastocyst stage do not displace the entire host epiblast, whereas only 8-cell stage injections result in fully ESC-derived F0 mice. To acknowledge this, we have amended the text mentioning this reference in the Introduction to the following: "If enough donor cells are injected, the resulting mouse can be composed entirely of donor cells; in contrast, injection at the blastocyst stage results in chimeras that are only partially derived from ESCs (Poueymirou et al. 2007)."

© 2025. Published by The Company of Biologists under the terms of the Creative Commons Attribution License (https://creativecommons.org/licenses/by/4.0/).

Minor

8. Fig. 1D: Difference between with and w/o feedback curves are hard to see.

To address this, we have increased the line thickness in **Fig. 1D** to improve visual clarity and better distinguish the curves with and without feedback.

9. The labels in Fig. 2C, D are hard to get - what is donor, what is host?

To address this, we have amended the coloured text labels under these figure panels from "Donor", "Host", and "PrE" to "Donor EPI", "Host EPI", and "PrE".

10. The authors write: "Together this suggests that donor cells are able to drive host cells into the TE compartment during the first cell-fate decision. In the instance where the donor cells are Fgf4-/-, host cells specify into either EPI or PrE." This is confusing - how can host cells still decide between Epi and PrE if they've been pushed to TE already?

We apologise for the lack of clarity here. To address this, we have amended this text to the following: "Together this suggests that donor cells, regardless of genotype, are able to drive host cells into the TE compartment during the first cell-fate decision. Subsequently, in chimeras with *Fgf4*$^{-/-}$ donor cells, remaining host cells that become ICM can then specify into either EPI or PrE. In contrast, in chimeras with *Fgf4*$^{+/+}$ donor cells, these remaining unspecified host ICM cells are biased to the PrE compartment, most likely by FGF4 signalling."

11. I am not sure the title ideally captures the main message of the manuscript - what the donor cells do is not so much impede epiblast specification, they rather displace host cells towards extraembryonic lineages.

To address this, we have amended the title to "Donor embryonic stem cells displace host cells of 8-cell stage chimeras to the extraembryonic lineages by spatial crowding and FGF4 signalling".

<u>Second decision letter</u>

MS ID#: dev.204518R1

MS TITLE: Donor embryonic stem cells displace host cells of 8-cell stage chimeras to the extraembryonic lineages by spatial crowding and FGF4 signalling

AUTHORS: Stanley E. Strawbridge; Anna Katharina Schrattel; Peter Humphreys; Ken A. Jones; Jérôme Artus; Anna-Katerina Hadjantonakis; Alexander G. Fletcher; Jennifer Nichols

Dear Dr Strawbridge,

I have now received all the referees reports on the above manuscript, and have reached a decision. The referees' comments are appended below, or you can access them online: please go to .

The overall evaluation is positive and we would like to publish a revised manuscript in Development, provided that the referees' comments can be satisfactorily addressed. Referee 1 has some suggestions to improve the clarity and accessiblity of the study. Please attend to all of the reviewers' comments in your revised manuscript and detail them in your point-by-point response. If you do not agree with any of their criticisms or suggestions explain clearly why this is so. If it would be helpful, you are welcome to contact us to discuss your revision in greater detail. Please send us a point-by-point response indicating your plans for addressing the referees' comments, and we will look over this and provide further guidance.

© 2025. Published by The Company of Biologists under the terms of the Creative Commons Attribution License (https://creativecommons.org/licenses/by/4.0/).

Reviewer 1

In their revised version, Strawbridge and colleagues have improve their manuscript and addressed some of our concerns.

We understand and respect the opinion of the authors and we would like to explicitly say that our concerns were simply stated in the interest of 1) the readability and 2) the completeness of the manuscript. We also want to acknowledge that we had incorrectly read the data in Fig 3l and we thank the authors for clarifying this to us. We got confused by the presentation of the statistical analyses.
We think that in its current state, the manuscript is not as accessible as it could be, which is unfortunate, and, more importantly, lacking key information that were provided in the response to reviewers but were not included in the manuscript.

Could the author include the statistical analyses in the manuscript, as a table for example? Having that key information on a separate repository whose sustainability is questionable is simply not acceptable.

Could the authors include the paragraph about the method of EScells injection in the methods section instead of simply referring to a previous study? In 20 years' time, readers will be happy not to have to dig further to understand how experiments were done.

Could the authors clarify in the method what objective criteria are used to define a cell as SOX2 or GATA4 positive? Do they measure nuclei intensity? Do they have a threshold value? Or is this manual curation by eye? Clarifying this is essential for future readers to understand and repeat those experiments.

Could the authors include the simulation with crowding but without differential growth? Without it the manuscript feels incomplete.

Reviewer 2

The authors have addressed all the comments from my initial report

Reviewer 3

SUMMARY OF THE ADVANCE MADE IN THIS PAPER AND ITS POTENTIAL SIGNIFICANCE TO THE FIELD

See my previous review

SUGGESTIONS TO AUTHORS

The authors have made a number of amendments and re-writes to the manuscript to address the extensive set of reviewer comments. These changes have much improved the manuscript, such that it is ready for publication in Development.

Minor
Fig. S1D is not cited in the text, please correct.

Second revision

Author response to reviewers' comments

Detailed response to reviews

© 2025. Published by The Company of Biologists under the terms of the Creative Commons Attribution License (https://creativecommons.org/licenses/by/4.0/).

We thank the anonymous reviewers for their constructive comments. Below, we provide a detailed point-by-point response outlining the changes made and our rationale. We hope that our revisions and responses address their concerns.

Reviewer 1 Comments for the Author:

In their revised version, Strawbridge and colleagues have improve their manuscript and addressed some of our concerns.

We understand and respect the opinion of the authors and we would like to explicitly say that our concerns were simply stated in the interest of 1) the readability and 2) the completeness of the manuscript. We also want to acknowledge that we had incorrectly read the data in Fig 3l and we thank the authors for clarifying this to us. We got confused by the presentation of the statistical analyses.

We think that in its current state, the manuscript is not as accessible as it could be, which is unfortunate, and, more importantly, lacking key information that were provided in the response to reviewers but were not included in the manuscript.

Could the author include the statistical analyses in the manuscript, as a table for example? Having that key information on a separate repository whose sustainability is questionable is simply not acceptable.

To address this point, we have duplicated the summary statistics and statistical test results underlying **Fig. 2E-G** and **Fig. 3G-I** from the GitHub repository as a new supplementary table, **Table S1**.

Could the authors include the paragraph about the method of EScells injection in the methods section instead of simply referring to a previous study? In 20 years' time, readers will be happy not to have to dig further to understand how experiments were done.

We unintentionally omitted in our initial response that we had, in fact, included the requested methodological details in the revised ***MATERIALS AND METHODS*** section under the subheading ***Embryo culture and chimera generation***. We thank the reviewer for this comment and agree that methodological clarity is essential for long-term accessibility. The revised section now provides a full description of the ESC injection procedure (including embryo staging, injection technique, and post-injection culture) without relying solely on prior references, and has been expanded in response to the reviewer's initial query.

Could the authors clarify in the method what objective criteria are used to define a cell as SOX2 or GATA4 positive? Do they measure nuclei intensity? Do they have a threshold value? Or is this manual curation by eye? Clarifying this is essential for future readers to understand and repeat those experiments.

As noted in our previous response, we updated the ***MATERIALS AND METHODS*** subsection ***Quantitative image analysis*** to include a more detailed description of the manual cell counting used for the data in **Fig. 2**. To address the reviewer's outstanding comment, we have further refined the description to explicitly clarify the objective criteria used for manual scoring. The revised passage now reads:

"Manual cell counting of ICMs from embryos and chimeras collected at E2.5+48 hrs was performed on confocal z-stacks using Fiji (ImageJ) with the 'Cell Counter' plugin. Individual nuclei were scored and assigned to lineages by eye, based on fluorescence marker expression. A nucleus was considered marker-positive if its signal was clearly above local background and showed nuclear enrichment for SOX2 and GATA4, or both nuclear and cytosolic enrichment for DsRed. SOX2-positive cells were classified as EPI, GATA4-positive cells as PrE, and cells double-positive for DsRed and SOX2 as donor-derived EPI."

Could the authors include the simulation with crowding but without differential growth? Without it

© 2025. Published by The Company of Biologists under the terms of the Creative Commons Attribution License (https://creativecommons.org/licenses/by/4.0/).

the manuscript feels incomplete.

We respectfully disagree with the reviewers' suggestion that the manuscript is incomplete without the inclusion of a model incorporating crowding without differential growth. As described in our previous response, our initial investigations revealed that this model exhibited substantial epiblast overgrowth that is inconsistent with experimental observations. These findings motivated our inclusion of differential growth in all subsequent models.

Empirical data from Saiz et al. (2016) estimate the net growth rate of the preimplantation embryo, from zygote (1 cell) to the E4.5 blastocyst (~150 cells), to be approximately 0.0669 h^{-1}. Our embryo models aligns with this, estimating a growth rate of ~0.06 h^{-1}. In contrast, using data from Alexandrova et al. (2016), we calculate that donor ES cells injected at the 8-cell morula stage exhibit a much slower growth rate of ~0.018 h^{-1} over the 48-hour window used in our experiments and modelling. Correspondingly, our chimera models incorporating differential growth estimate the donor cell growth rate at ~0.02 h^{-1}.

These differences support the need for differential growth in the model, and we emphasise this point explicitly in the manuscript. However, since these empirical relationships may not be familiar to all readers, our modelling approach was deliberately constructed in a stepwise fashion to guide the reader through increasingly refined models. This narrative demonstrates how differential growth emerges as a necessary condition to explain the observed dynamics.

We therefore chose not to include the crowding-only model in the manuscript, as doing so would interrupt the logical flow and distract from the mechanistic insight provided by the models that better match the data.

Reviewer 2 Comments for the Author:

The authors have addressed all the comments from my initial report
We thank the reviewer for their constructive feedback.

Reviewer 3 Comments for the Author:

SUMMARY OF THE ADVANCE MADE IN THIS PAPER AND ITS POTENTIAL SIGNIFICANCE TO THE FIELD

See my previous review SUGGESTIONS TO
AUTHORS
The authors have made a number of amendments and re-writes to the manuscript to address the extensive set of reviewer comments. These changes have much improved the manuscript, such that it is ready for publication in Development.

We thank the reviewer for their constructive feedback.

Minor

Fig. S1D is not cited in the text, please correct.

We thank the reviewer for catching this typographical oversight. The sentence in question has been corrected to read:

"To capture intrinsic stochasticity in small cell populations, we ran Gillespie simulations using the median posterior parameter values from the PrE feedback model (Fig. S1D)."

We have also previously referenced **Fig. S1D** in the ***Data Availability*** section and have carefully checked the manuscript to ensure that all figure panels are now appropriately cited in the main text.

© 2025. Published by The Company of Biologists under the terms of the Creative Commons Attribution License (https://creativecommons.org/licenses/by/4.0/).

Third decision letter

MS ID#: dev.204518R2

MS TITLE: Donor embryonic stem cells displace host cells of 8-cell stage chimeras to the extraembryonic lineages by spatial crowding and FGF4 signalling

AUTHORS: Stanley E. Strawbridge; Anna Katharina Schrattel; Peter Humphreys; Ken A. Jones; Jérôme Artus; Anna-Katerina Hadjantonakis; Alexander G. Fletcher; Jennifer Nichols
ARTICLE TYPE: Research Report

Dear Dr Strawbridge,

I am happy to tell you that your manuscript has been accepted for publication in Development, pending our standard publication integrity checks.

© 2025. Published by The Company of Biologists under the terms of the Creative Commons Attribution License (https://creativecommons.org/licenses/by/4.0/).
